# Diagnostics and therapy of sudden hearing loss

**DOI:** 10.3205/cto000144

**Published:** 2018-02-19

**Authors:** Stefan K. Plontke

**Affiliations:** 1 Department of Otorhinolaryngology, Head & Neck Surgery, University Medicine Halle, Martin Luther University Halle-Wittenberg, Halle (Saale), Germany

**Keywords:** sudden hearing loss, differential diagnosis, intralabyrinthine schwannoma, intratympanic

## Abstract

This article reviews recent aspects of diagnostics, differential diagnostics, and evidence in systemic and local therapy of idiopathic sudden sensorineural hearing loss (ISSHL). Since a number of disorders can be accompanied by sudden hearing loss, a meaningful and targeted diagnostic strategy is of utmost importance. An important differential diagnosis of sudden hearing loss are intralabyrinthine schwannomas (ILS). The incidence of ILS is probably significantly underestimated. This may be due to the lack of awareness or lack of explicit search for an intralabyrinthine tumor on MRI or an inappropriate MRI technique for the evaluation of sudden hearing loss (“head MRI” instead of “temporal bone MRI” with too high slice thicknesses). Therefore, the request to the radiologist should specifically include the question for (or exclusion of) an ILS. With special MRI techniques, it is possibly today to visualize an endolymphatic hydrops. The evidence in the therapy of ISSHL is – with respect to the quality and not quantity of studies – unsatisfying. The value of systemically (low dose) or intratympanically applied corticosteroids in the primary treatment of ISSHL is still unclear. In order to investigate the efficacy and safety of high dose corticosteroids as primary therapy for ISSHL, a national, multicenter, three-armed, randomized, triple-blind controlled clinical trial is currently performed in Germany (http://hodokort-studie.hno.org/). After insufficient recovery of the threshold with systemic therapy of ISSHL, intratympanic corticosteroid therapy appears to be associated with a significantly higher chance of an improved hearing threshold than no therapy or placebo. Both, hearing gain and final hearing threshold, however, appear to be independent from the onset of secondary therapy. Based on currently available data from clinical studies, no recommendation can be made with respect to the type of corticosteroid and specifics of the intratympanic application protocol.

## 1 Introduction

According to the WHO, about 360 million people suffer from hearing impairment worldwide. In the European Union, the number is expected to amount to 434,000 people suffering from deafness and 44,000,000 people suffering from hearing impairment. A recent epidemiological investigation on the hearing status (HÖRSTAT) in Germany revealed a prevalence of hearing loss based on the WHO classification of about 16% [1]. 

In the WHO list entitled “Global Burden of Disease”, hearing impairment ranks 15^th^, and 2^nd^ regarding the “Disability-adjusted life years”. The majority of patients affected by hearing loss (>80%) suffer from sensorineural hearing loss. Apart from age-associated, drug-induced as well as noise-induced hearing loss, sudden idiopathic sensorineural hearing loss is the most frequent cause.

### 1.1 Definition

According to the AWMF guidelines, sudden hearing loss (ISSHL) is a suddenly appearing, generally unilateral hearing loss of cochlear origin with unknown cause (i.e. idiopathic), expressing different degrees of severity up to complete deafness (anacusis). Vertigo and/or tinnitus may appear additionally [2].

### 1.2 Incidence

The incidence of sudden hearing loss in the population of the industrialized nations was estimated to 5–20 per 100,000 inhabitants [3]. More recent investigations, however, allow the assumption that the incidence is much higher with 160 [4] or possibly even up to 400 per 100,000 inhabitants [5]. The mean age of the patients being included in randomized controlled clinical trials (RCT) amounts to 45–55 years. Males and females are affected equally. In childhood, sudden idiopathic hearing loss occurs very rarely [2].

## 2 Diagnostics

Acute hearing loss (hearing impairment) may be due to many different causes. The bases of the differential diagnostic decision tree are the patient’s history (if needed obtained by a third party), ear microscopy, tuning fork tests according to Weber and Rinne, and pure tone audiometry followed by targeted audiological, neuro-otological, imaging, and further diagnostics [6], [7].

First, causes of sudden hearing loss such as pathologies of the external auditory meatus and the middle ear as well as severe systemic diseases have to be excluded of which acute hearing loss is only an accompanying symptom, as for example cardiovascular emergencies (e.g. hypertensive crisis) or neurological emergencies (e.g. stroke).

The identification of pathologies affecting the middle ear or the auditory meatus (including ceruminal plugs) is performed by ear microscopy and tuning fork tests.

The current version of the German S1 guideline on ISSHL recommends the following elements as necessary diagnostics (translated from: [2]):

- “Intensive general and specific history taking
- ENT-specific physical examination
- Blood pressure measurement
- Ear microscopy
- Hearing tests (tuning fork, pure tone audiogram)
- Tympanometry
- preliminary vestibular testing”

Hereby, it appears to be reasonable to precisely define the term of “preliminary vestibular testing” in a revised version of the guideline.

In the last version of the German S1 guideline on ISSHL, the following procedures are recommended as useful in individual cases [2]. In this context, it seems to be required or suitable to determine a more detailed indication and evidence assessment of single diagnostic measures in a revised version of the guideline.

- “Otoacoustic emissions (OAE)
- Auditory evoked brainstem potentials (ABR)
- Speech audiometry
- Stapedius reflex measurement
- Functional examination of the cervical spine
- Laboratory tests: blood glucose, CRP, procalcitonin, small blood count, differential blood count, creatinine, fibrinogen level
- Serologic testing: borreliosis, syphilis, herpes simplex virus type 1, varicella zoster virus, CMV, HIV
- MRI: exclusion of a tumor of the cerebellopontine angle (hearing protection is recommended)
- CT scan: skull, temporal bone, cervical spine
- Glycerol test according to Klockhoff
- Electrocochleography: cochlear damage, exclusion of hydrops
- CERA: exclusion of psychogenic deafness
- Auditory steady state responses (ASSR)
- Electronystagmography or video-oculography
- Duplex sonography
- Tympanoscopy
- Interdisciplinary examinations (e.g. neurology, internal medicine, orthopedics, human genetics)”

When planning extended diagnostics, noise exposure of the inner ear (for example due to measurement of the stapedius reflex and acoustically evoked potentials, magnet resonance imaging) should be avoided, if possible, during the first days after acute hearing loss occurred. 

### 2.1 Vestibular tests

In cases of acute hearing loss in the context of an acute vestibular syndrome, i.e. if vertigo and/or nystagmus are observed, the following questions should always be clarified in the clinical examination to exclude a stroke (partly translated from [8]):

1. Is a catch-up saccade observed in the clinical head impulse test for the horizontal vestibulo-ocular reflex (hVOR) and does it correspond to the affected side [9]?
2. Are eye movement disorders and nystagmus observed?
3. Is skew deviation observed?

In 2009, Kattah et al. [10] introduced the term HINTS (head impulse test, nystagmus, test of skew). The combination of those 3 vestibular screening examinations (1. Clinical head impulse test for hVOR, 2. Eye movement analysis (nystagmus), and 3. The diagnosis of a vertical divergence of the eyes (skew deviation)) has a higher sensitivity and specificity in the early phase of acute vestibular symptoms regarding stroke than a diffusion-weighted MRI (Table 1 (Tab. 1)).

Brandt et al. [11] recommend a 5-step procedure of the clinical examination to exclude stroke:

1. Testing for skew deviation
2. Differentiation of a peripheral spontaneous nystagmus and a central fixation nystagmus by means of fixation suppression and Frenzel goggles
3. Examination of a gaze-evoked nystagmus (central disorder) in the opposite direction of the spontaneous nystagmus
4. Examination of saccadic smooth pursuit
5. Performance of a clinical head impulse test for the hVOR.

Thus, the authors achieve also a high sensitivity and specificity in the differential diagnosis of stroke. If HINTS and/or the five procedures suggested by Brandt et al. do not indicate a central cause, a peripheral origin is very probable.

### 2.2 Otoacoustic emissions (OAE)

Transient-evoked otoacoustic emissions and distortion product otoacoustic emissions are of general relevance for the evaluation of sensory hearing loss [12]. The presence of OAE in measurements early after ISSHL seems to show a good prognosis for hearing recovery [13], [14], [15].

### 2.3 Speech audiometry

To assess the functional deficit after ISSHL, speech audiometry is more important than pure tone audiometry. Regarding speech audiometry, the Freiburg monosyllabic test in quiet [16], [17] at fixed levels of 65 and 80 dB SPL is recommended. After an interval, testing of the maximally achievable percentage of understanding monosyllables in quiet is significant for the evaluation of the possibility and the success of hearing aid fitting. In general, speech tests in noise are more relevant regarding speech understanding and thus for communication in everyday situations. They thus have the highest relevance for the patients, but they are not yet established as standard procedures on a national or international basis [18], [19], [20].

### 2.4 Auditory evoked brainstem responses (ABR)

The measurement of auditory evoked brainstem responses (ABR) provides information on the retrocochlear auditory pathways. Due to their direct neighborhood to the cochlear nerve in the internal auditory meatus, vestibular schwannomas (VS), also referred to as ”acoustic neuromas” (AN), that most frequently have their origin in the superior and inferior vestibular nerves, lead to an impairment of the conduction of action potentials in the auditory nerve. This results in a relatively or absolutely prolonged latency of the ABR waves JIII and JV generated in the brainstem during acoustic stimulation with click stimuli, when the two sides are compared. In order to exclude systematic errors, the latency difference of the waves JIII and JV is measured in relation to the latency of the wave JI generated in the cochlea.

Since the late 1970ies, the measurement of the ABR has been established for the diagnosis of VS [21], [22]. From the late 1980ies on, MRI measurement with its higher sensitivity for smaller tumors in comparison to ABR measurement could be established as the gold standard [23], [24]. Especially in the context of follow up for VS after conservative therapy (“wait-and-test-and-scan”), associated with questions of cost efficiency of the examination methods, ABR measurements remain in the methodological inventory.

A meta-analysis evaluated 43 trials of 623 screened studies on the application of ABR for diagnosis of VS – from 1978 to 2009 [25]. However only patients were included that had an intraoperative confirmation of VS. The pooled sensitivity of ABR amounted to 93.5% for 3,314 patients (confidence interval: 92.6–94.3%). Thus, there is a false-negative rate of 6.5%, which in this context corresponds to 216 tumors that were not detected by ABR. For tumors smaller than 1 cm, the sensitivity even amounted to 85.8% (false-negative rate of 14.2%), for larger tumors it was 95.6%. This means that 1 of 7 tumors smaller than 1 cm would not be discovered by ABR. In a subgroup of the analyzed trials, also the specificity could be determined. In the meta-analysis, it amounted to 82.0% (confidence interval: 80.5–83.6%), which corresponds to a false-positive rate of 18.0%. Those parameters are relatively constant over the high number of analyzed trials.

These data, that are consistent over many trials, show that the ABR measurement has a high selectivity for the diagnosis and follow-up of VS. This also applies when a possible selection bias within the evaluated studies for large tumors and actual surgical intervention is considered. In practice, a false-negative rate of 14.2% means that with an incidence of VS in patients with asymmetric hearing loss of 1–7% [25], 0.55% of all patients with asymmetric hearing loss (1 in 200) and every 7^th^ patient with an actually present VS had the risk of a tumor that was not detected by ABR measurement. On the other hand, a false-positive rate of 18.0% shows that some patients would still undergo unnecessary MRI examination.

Nonetheless, it must be mentioned that also patients that observe complete hearing recovery after ISSHL, should undergo MRI because even complete hearing recovery (spontaneous or for example after systemic corticosteroid administration) does not exclude vestibular schwannoma (acoustic neuroma) [26], [27].

Progress and development of electrophysiological diagnostics may further increase the sensitivity and specificity of VS diagnostics. For example, apart from measurements at supra-threshold levels, also registrations at 40 dB SL can be useful to differentiate cochlear and retrocochlear lesions. For patients with good hearing, those measurements could be the basis of intraoperative monitoring in cases of small VS in order to better evaluate possible level-related changes of the auditory nerve [28].

Both methods, MRI and ABR, have their limitations and cannot be applied in all patients. On the one hand, the number of patients with contraindications or restrictions for MRI examinations continuously increases due to the high number of implants with limited MRI-compatibility. On the other hand, ABR measurements cannot or only to a limited extent be applied in patients with additional conductive hearing loss, because hereby the wave JI cannot be registered. MRI, however, provides information that go beyond the presence of VS which is not the case for ABR measurements. The question of cost efficiency will be answered also depending on different health systems in different countries. Both methods should be available as complementary procedures. This is useful for the preservation of the expertise and allows investigations of all patients despite possible, individually contraindications for one of the two methods.

### 2.5 Laboratory examinations

Specific laboratory examinations (clinical chemistry, serologic testing) may support the differential diagnostics of acute hearing loss. The existing evidence regarding the usefulness of laboratory examinations is based only on case series and small case control studies without sufficiently demonstrating their benefit. Routine examinations, however, are not recommended. In this context, “routine” refers to untargeted tests that are automatically performed or to test batteries without taking into account the specific characteristics of the patient and his concomitant diseases, additional symptoms, or anamnestic and geographic risk factors [29].

### 2.6 Magnetic resonance imaging

Magnetic resonance imaging of the head and the temporal bone with contrast enhancement performed with special attention to the inner ear is generally indicated in all patients showing neuro-otological symptoms, in particular if unilateral and in every patient with acute idiopathic, sensorineural hearing loss. Usually, examination after an interval is sufficient. An immediate diagnostic is rarely indicated and should be performed only when a neurological emergency situation is expected. Even complete recovery of the hearing threshold after ISSHL does not exclude the cause of a pathology that may be diagnosed by MRI [26], [27].

In the context of an own investigation, 198 magnetic resonance images were carefully described that had been performed for diagnostics of patients with acute, sensorineural severe to profound hearing loss (>70 dB HL) or with acute deafness (anacusis, i.e. no measurable threshold). The variety of pathological findings along or near the auditory pathways confirms the importance that not only vestibular schwannoma has to be excluded in the context of diagnostics of sudden hearing loss, but that the complete auditory pathway from the inner ear to the superior temporal gyrus must be screened by means of magnetic resonance imaging. In this MRI study, 83 patients (41.9%) revealed pathological findings that were related to acute hearing loss. In the other 115 patients (58.1%) with sudden hearing loss, the MRIs were without pathological findings with respect to the auditory pathways. There was no correlation between the severity of sudden hearing loss and the probability of pathological MRI findings [30]. In other, partly larger studies, however, without special attention to cases with severe or profound sudden hearing loss, various frequencies of pathological MRI results were described (Table 2 (Tab. 2)).

### 2.7 Computed tomography

**Figure 1:**
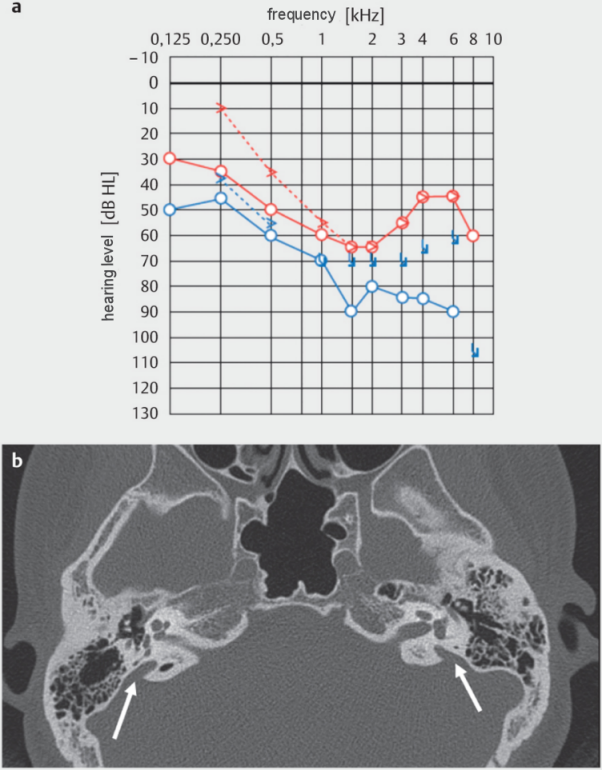
Acute sensory hearing loss after minor trauma in a patient with large vestibular aqueduct syndrome (LVAS; syn.: large endolymphatic duct syndrome or large endolymphatic duct and sac (LEDS); arrows); a: pure tone audiometry (red: right side; blue: left side); b: computed tomography (native, axial); Department of Radiology, University Medicine Halle, courtesy of Prof. S. Kösling.

Patients with sudden hearing loss, i.e. without any hint for a possible cause of the acute sensorineural hearing loss after history taking and examination, are not recommended to undergo immediate computed tomography for differential diagnosis because of the low significance, the costs, and radiation exposure [29]. If a malformation of the inner ear is suspected (for example large vestibular aqueduct syndrome, LVAS; Figure 1 (Fig. 1)), computed tomography is suitable [31], [32].

### 2.8 Tympanoscopy

Tympanoscopy with sealing of the round window membrane (and the oval window, if needed) to close a “perilymph fistula” is often advocated as treatment for sudden hearing loss, especially for profound sudden hearing loss or acute deafness (anacusis without measurable hearing threshold) and vertigo. This practice is nearly exclusively based on case series which assumed, that the observed changes of hearing threshold were due to the intervention, although they were lacking comparison with an adequate control group [33], [34], [35], [36], [37]. In this respect, certain anatomical and surgical aspects must be considered. Only rarely, the round window membrane can be sufficiently examined by microscopic inspection of the middle ear. Most of the studies did not mention reduction of the “promontorial overhang” in order to completely visualize the round window membrane, which probably means that this has not been performed. Furthermore, there are often so-called “false round window membranes” that may obliterate the round window niche completely or partially and can be misinterpreted as “ruptures of the round window membrane” [38], [39]. 

Especially in the context of clinical studies where the surgeons selects the intervention and also interprets the intraoperative findings, randomization (neither the patient nor the therapist/surgeon have an influence on the assignment to the treatment arm) and blinding at least of the endpoint measurements are crucial.

In summary, the hypothesis of the efficacy of tympanoscopy with sealing of the round window membrane for treatment of idiopathic sudden hearing loss (in contrast to barotrauma, stapes surgery, or temporal bone trauma) can neither be confirmed nor rebutted on the basis of currently available data [40], [41]. 

## 3 Differential diagnostics

There is a high number of diseases that are characterized by the symptom of acute unilateral sensorineural hearing loss (Table 3 (Tab. 3)).

Therefore, it is important to look for anamnestic and clinical signs that make a definable cause of acute sensorineural hearing loss (hearing impairment) probable. Those are hints to other otological, or cardio-vascular, endocrinological, metabolic, (auto)immunological, neurological, and other diseases. 

Whenever a cause for the acute sensorineural hearing loss can be identified or is considered probable, it should not be termed “sudden (idiopathic) hearing loss”; e.g. acute sensorineural hearing loss due to an ipsilateral tumor of the cerebellopontine angle (vestibular schwannoma) should not be called “sudden (idiopathic) hearing loss”. The hearing loss (hearing impairment) should then be further examined and treated based on the guidelines of the identified disease or the “working diagnosis”.

Such typical anamnestic data, symptoms, or hints might be for example:

1. sudden bilateral hearing loss (which is rarely idiopathic),
2. recurrent sudden or fluctuating hearing loss,
3. isolated hearing loss in the lower frequencies,
4. simultaneous, bilateral vestibular disorders with oscillopsia,
5. accompanying focal neurological deficits such as pareses, dysarthria, ataxia, encephalopathy, headache, diplopia,
6. vertical or gaze-evoked nystagmus and skew deviation,
7. pathological findings of the central nervous system in the imaging,
8. head trauma with timely relationship to the hearing loss, or
9. ophthalmological symptoms.

Bilateral hearing loss that appears suddenly or develops over a very short time has often vascular, metabolic, (auto)immunological, infectious, neoplastic, chemical toxic, or inflammatory causes, even if the bilateral occurrence is not pathognomonic for these causes. Also unilateral symptoms can be observed with those findings. Possible systemic causes for sudden bilateral sensorineural hearing loss must be clarified promptly [42].

**Figure 2:**
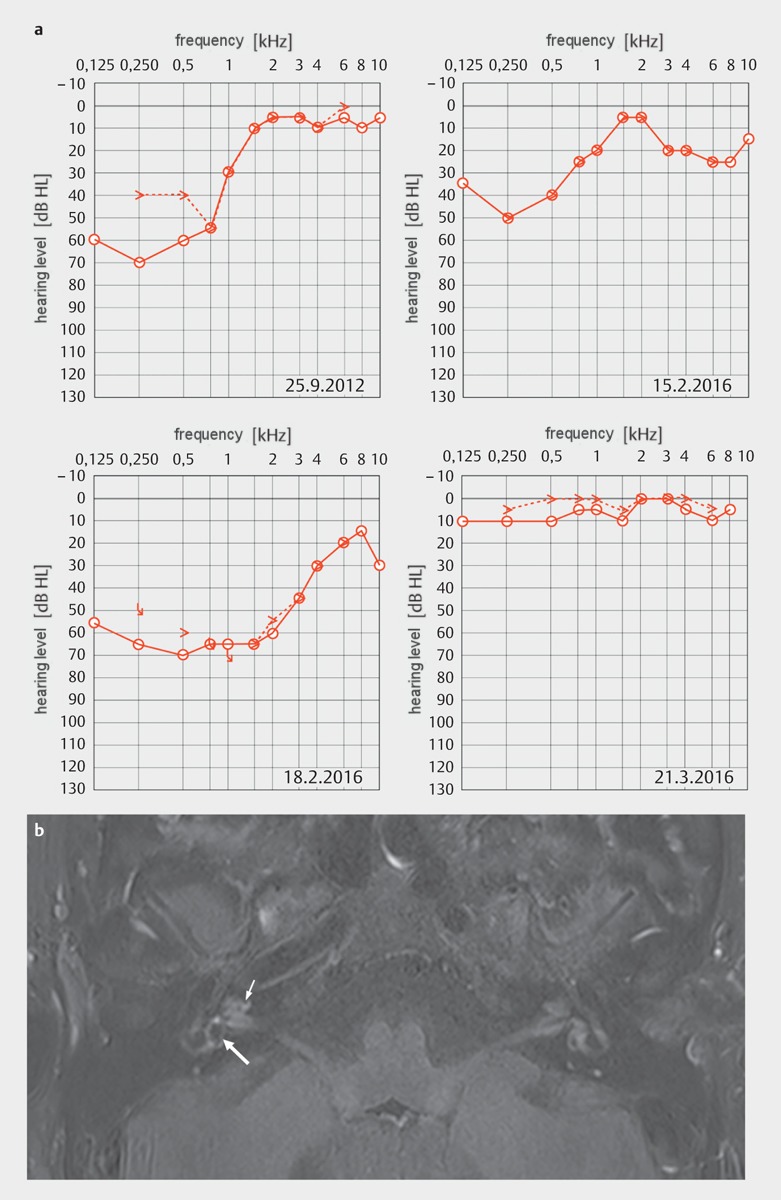
Hydropic ear disease, right-sided: a: The patient suffered from “recurrent sudden hearing losses” in the lower frequency range on the right side, which recovered during systemic high-dose prednisolone therapy (“fluctuating hearing loss”). Considering the imaging (b), the disease is classified as “primary hydropic ear disease of cochlear type” and when vertigo appears as “primary hydropic ear disease of the cochlea-vestibular type” or Menière’s disease [46]. b: MRI reveals an enlarged endolymphatic space in the cochlea (small arrow) and in particular in the vestibulum (large arrow) of the right side, indicating a moderate endolymphatic hydrops (3D IR sequence, 6 hours after intravenous application of contrast medium); CM: contrast agent; w: weighted. (b Department of Radiology, University Medicine Halle, courtesy of Prof. Dr. S. Kösling).

Fluctuating hearing loss or hearing loss in the low frequencies, often perceived (and treated) as recurrent sudden hearing loss, allow the assumption of hydropic ear disease of cochlear origin (see chapter on hydropic ear disease) (Figure 2 (Fig. 2)) or – if it occurs together with characteristic episodes of vertigo – Menière’s disease, an (auto)immunological disease, Cogan syndrome, or hyperviscosity syndrome [43], [44], [45], [46]. 

Some differential diagnoses (see Table 3 (Tab. 3)) will be separately described in the following paragraphs.

### 3.1 Vestibular schwannoma (acoustic neuroma) 

Vestibular schwannoma (acoustic neuroma), especially also with intralabyrinthine location, is an important and relatively frequent differential diagnosis of sudden hearing loss. In an own patient population, vestibular schwannoma was diagnosed in 8 of 198 (4%) of patients with sudden hearing loss by means of MRI. Although only patients with acute severe to profound hearing loss (mean hearing loss in the frequency ranges of 0.5–4 kHz >70 dB HL) or acute deafness were included in this study, a literature analysis did not show higher rates of vestibular schwannoma compared to patients with less severe idiopathic sudden hearing loss [30]. 

The incidence of vestibular schwannomas detected by MRI in patients with ISSHL varies in the literature. The mean value of the studies displayed in Table 4 (Tab. 4) amounted to 3.9% (median: 3.8; standard deviation: 1.6; 95% confidence interval: 28.–5.0). Since the percentage of 4% in our study [30] is within the range of the 95% confidence interval of the studies described in Table 4 (Tab. 4), the difference is not significant.

Even if a vestibular schwannoma is present, treatment as for ISSHL, e.g. with systemic corticosteroids, may lead to complete recovery of the hearing threshold after acute (and assumed idiopathic) sudden hearing loss [26], [27]. 

### 3.2 Intralabyrinthine schwannomas (ILS)

Because of their location in the inner ear, intralabyrinthine schwannomas (ILS) appear to be a distinct entity and a rare but important differential diagnosis of ISSHL. Nearly all ILS cause an ipsilateral hearing loss as first symptom, which may be progressive, acute, or fluctuating, but usually of sensorineural nature [47]. ILS can also be accompanied by symptoms that are typical for hydropic ear disease [48], [49]. 

**Figure 3:**
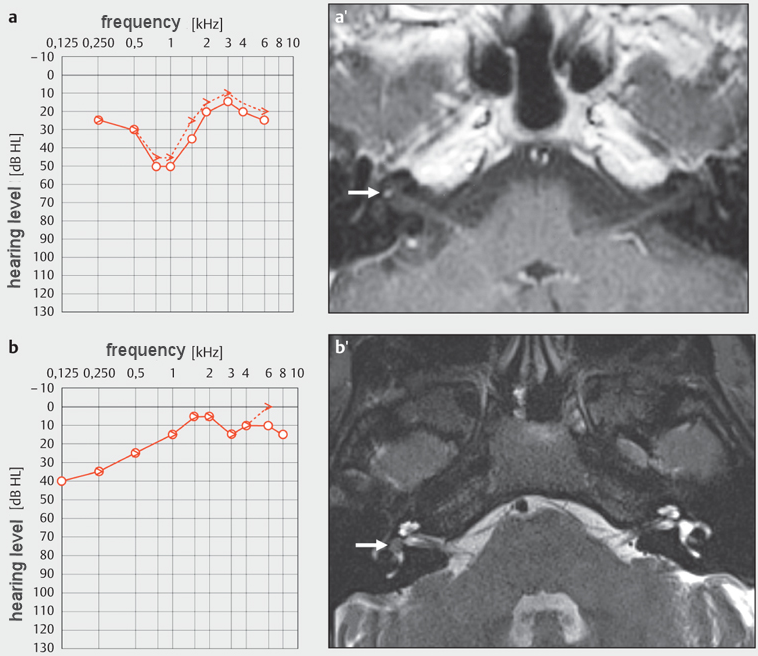
Intralabyrinthine schwannomas (a intracochlear; b intravestibular) with symptoms of sudden hearing loss: a, a’: Because of the symptoms of sudden hearing loss with mild to moderate medio-cochlear hearing loss, a MRI (axial, T1-w, contrast agent) had been performed in 2005. It showed a very small contrast enhancing mass in the right cochlea (arrow). In the course of 10 years, the tumor showed a progressive growth until it filled the whole cochlea. It was removed via subtotal cochleoectomy with partial reconstruction of the cochlea and insertion of a CI electrode dummy. The functions of the semicircular canals were preserved [53]. b, b’: Intravestibular schwannoma in the vestibule (arrow, T2-w, axial) with Menière’s disease like complaints and mild acute hearing loss in the lower frequency range. After increasing vertigo, the tumor was resected via labyrinthectomy and in the same session hearing rehabilitation was performed with cochlear implantation. CM: contrast medium; w: weighted. (b’: Department of Radiology, University Medicine Halle, courtesy of Prof. Dr. S. Kösling).

**Figure 4:**
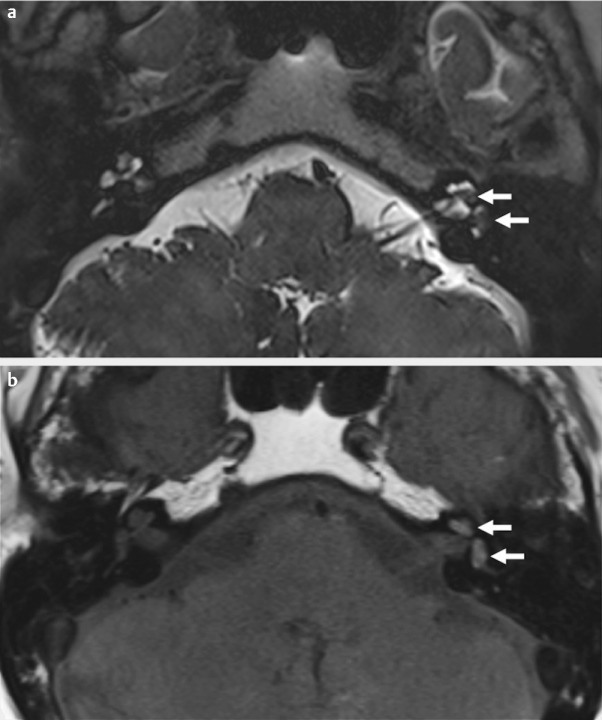
Spontaneous labyrinthine bleeding in the left inner ear. Acute left-sided deafness and vertigo of a patient after therapy for Non-Hodgkin-lymphoma of the central nervous system (Ann Arbor stage IV) 3 years before and drug-induced coagulopathy (anticoagulation medication) due to port thrombosis. In the area of the cochlear as well as the vestibular part of the inner ear, MRI revealed punctually clear signal decreases (arrows) in the T2-weighted (a) and a minor signal increase (arrows) in the native T1-weighted images (b). w: weighted. Department of Radiology, University Medicine Halle, courtesy of Prof. S. Kösling.

According to their location, ILS are classified into intracochlear, intravestibular, combined cochlea-vestibular, transmodioloar and transmacular (extension from the cochlea or the vestibulum into the internal auditory meatus), multilocular, and transotic tumors (Figure 3 (Fig. 3), Figure 4 (Fig. 4)) [50], [51], [52]. 

The incidence of intralabyrinthine schwannomas is probably underestimated. If they are missed, this is often due to the small size at the time of first occurrence of symptoms [53], due to an MRI that is inappropriate for the diagnosis (high slice thickness, MRI of the head instead of the temporal bone) and a lack of explicit attention to a possible intralabyrinthine tumor. Currently, the gold standard for the diagnosis is a thin-layer MRI of the temporal bone with contrast medium where tumors show a clear enhancement in the T1 weighted contrast-enhanced sequence and a missing fluid signal in thin-layer T2 weighted sequences [51], [54], [55], [56], (Figure 3 (Fig. 3)). 

Requesting an MRI examination for sudden hearing loss should especially encompass the question to the radiologist of excluding an intralabyrinthine schwannoma. Cochlear implantation together with surgical removal of an ILS is an option for auditory rehabilitation in these cases. If ILS are diagnosed early, it might be a promising therapeutic approach in contrast to a wait-and-test-and-scan strategy. Radiotherapy is only indicated in very limited cases, e.g. for tumors with transmodiolar growth and extension into the internal auditory canal in older patients without vertigo [57], [58].

### 3.3 Large vestibular aqueduct syndrome

The large vestibular aqueduct syndrome (LVAS) (synonym: LEDS - large endolymphatic duct syndrome or large endolymphatic duct and sac) is observed as an isolated malformation or in combination with other malformations such as for example Mondini malformation, Branchio-Oto-Renal syndrome, or Pendred syndrome. Hearing loss is generally fluctuating and often progresses in stages. Thus, it may be confused with a recurrent (progressive) idiopathic sudden hearing loss. Acute deterioration of hearing is also typical for minor trauma of the head (Figure 1 (Fig. 1)). Even if the malformation often appears on both sides, the symptoms are frequently asymmetric [31], [32], [59], [60]. A very rare differential diagnosis is a tumor of the endolymphatic sac, which occurs for example in von-Hippel-Lindau disease [61].

### 3.4 Hydropic ear disease

Hydropic ear disease comprises the spectrum of clinical manifestations of endolymphatic hydrops and must be differentiated from sudden hearing loss. The difference is made between the primary type (PHED, primary hydropic ear disease) and the secondary type (SHED, secondary hydropic ear disease). Furthermore, symptoms may be of cochlear, vestibular, or cochlea-vestibular nature. Hence, Menière’s disease with the fully developed symptom triad corresponds to PHED of the cochlea-vestibular type while fluctuating low frequency hearing loss corresponds to PHED of the cochlear type. Also, the Menière-like clinical entity, for example of LVAS or “delayed endolymphatic hydrops” is described by this terminology (SHED). On the other hand, confusing terms such as “atypical Menière’s disease”, “monosymptomatic Menière’s disease”, “cochlear Menière’s disease”, “forme fruste” should be avoided [9], [46], [48]. With modern MRI procedures, the endolymphatic hydrops can meanwhile be visualized in vivo in patients (Figure 2 (Fig. 2)) [62], [63].

### 3.5 Occlusion of the anterior inferior cerebellar artery (AICA syndrome)

The labyrinthine artery supplying the inner ear is most frequently a branch of the anterior inferior cerebellar artery (AICA). It is the artery of the three cerebellar arteries with the smallest caliber and originates from the basilar artery. Its supply region encompasses a part of the anterior cerebellar hemispheres and the lateral pons. Arteriosclerosis, vascular dissection, or thrombosis may lead to infarctions in the supply area of the AICA. In most of the cases of AICA occlusion, the symptom complex may also include unilateral hearing loss and vestibular disorders (nystagmus, vertigo) beside other symptoms like Horner’s syndrome, diplopia, facial paresis and dysesthesia, dysarthria, ataxia, nausea and vomiting, and contralateral reduction of pain and temperature sensation in different combinations. Those peripheral cochlea-vestibular disorders may also occur as isolated or prodromal symptoms [64]. Some cases were reported, in which exclusively unilateral hearing loss with tinnitus without cerebellar or brainstem-related symptoms were observed [65].

## 4 Systemic therapy

A large number of different drugs have been suggested and used for treatment of ISSHL. An extensive overview is found for example in [66]. In the following, a selection of the currently used therapeutic approaches will be described.

### 4.1 Corticosteroids

The rational basis for the treatment of acute cochleo-vestibular disorders with corticosteroids is their effect on glucocorticoid and mineral receptors found in the inner ear [67]. The protective effect of corticosteroids was confirmed in animal models for different traumas:

1. acute acoustic trauma [68], [69], [70], [71], [72];
2. amino-glycoside and cisplatin ototoxicity [73], [74];
3. pneumococcal meningitis [75];
4. autoimmune-associated hearing loss [76], [77];
5. insertion trauma in cochlear implantation (overview in [78]).

The treatment of sudden hearing loss with systemic standard dose corticosteroid therapy has been investigated in numerous trials of different, mostly low level evidence and significant amount of bias (overview in [66]). On the basis of those studies and the few RCTs (randomized controlled trials), the authors concluded in their meta-analysis that there was “no evidence of benefit of steroids over placebo” and “also no difference in the addition of antiviral therapy to systemic steroids, nor was there a difference between systemic steroids and other active treatment” (cited from: [79]). This aspect was confirmed by other review articles [80]. The authors of a Cochrane review on the systemic therapy of sudden hearing loss summarized: "The value of steroids in the treatment of idiopathic sudden sensorineural hearing loss remains unclear since the evidence obtained from randomized controlled trials is contradictory in outcome, in part because the studies are based upon too small a number of patients” [81]. Despite this conclusion, systemic steroids are applied in low or moderate doses (mostly 60 mg/d for about 10 days and afterwards daily dose reduction) worldwide as standard for primary therapy of sudden hearing loss [29], [66]. The administration of high-dose corticosteroids for the treatment of ISSHL is recommended by the German guideline on ISSHL [2] and performed in clinical routine practice in Germany.

The rational basis for the treatment of idiopathic sudden sensorineural hearing loss (ISSHL) with systemic high-dose steroids are retrospective cohort studies. Alexiou et al. analyzed the audiograms of 603 patients with sudden hearing loss; 301 of them (from 1986 to 1991) did not receive steroids and 302 patients (from 1992 to 1998) were treated with high-dose intravenously administered steroids (prednisolone) [82]. A treatment advantage was observed in the group of patients who underwent high-dose steroid therapy. Because of the study design, however, the bias of this trial is rather high. Egli Gallo et al. retrospectively investigated the efficacy of systemic high-dose therapy with dexamethasone (oral) and found a significant larger improvement of hearing compared to a historical control group that received the former standard therapy (oral medium dose prednisone therapy) [83]. Westerlaken et al. conducted a randomized clinical trial and did not find an advantage of super-high-dose steroid therapy compared to standard dose prednisolone [84]. Niedermeyer et al. showed that the cortisol level in the inner ear was only increased after intravenous application of 250 mg prednisolone and not with 125 mg i.v. [85]. In the German AWMF guideline on sudden hearing loss [2] the application of high-dose steroids (250 mg prednisolone or an equivalent steroid dose) is recommended as primary therapy of sudden hearing loss even if the benefit, as already mentioned, is not yet confirmed by randomized controlled clinical trials (RCTs).

This evidence gap is supposed to be closed in the context of a multicenter national clinical trial entitled “Efficacy and safety of high dose glucocorticosteroid treatment for idiopathic sudden sensorineural hearing loss – a three-armed, randomized, triple-blind, multicenter trial (HODOKORT: **HO**ch-**DO**sis-Gluko**KORT**ikoidtherapie)”. Hopefully, the clinical insecurity of the value of high-dose corticosteroid therapy in routine practise will disappear (see chapter on the necessity of clinical trials) [86]. 

In addition, the continuous reduction (“tapering out”) of corticosteroid dose after only few days (e.g. 5 days) of medium or high-dose application does not seem to be justified on the basis of current scientific knowledge, and lacking evidence, respectively.

### 4.2 Hyperbaric oxygenation

Hyperbaric oxygen therapy for treatment of sudden hearing loss was analyzed and evaluated in several review articles in similar ways and summarized as a potentially effective procedure. However, restrictions in the interpretation of the results arise from the relatively low number of patients as well as the procedure-related (referring to hyperbaric oxygenation) and methodical (referring to the study design) flaws. Thus, the results must be interpreted with care. Because of the costs and the possible undesired side effects, and at the same time insufficient evidence, the estimation of the benefit of hyperbaric oxygenation for the treatment of sudden hearing loss seems to be difficult [2], [29], [87], [88].

Based on the current data and due to new knowledge from investigations of combined intratympanic corticosteroid therapy with hyperbaric oxygenation [89], [90], [91], [92], a systematic, methodical high-quality investigation of the efficacy of this procedure would be desirable. Based on available experience, patients younger than 60 years, who still suffer from severe to profound hearing loss after unsuccessful primary therapy would be a suitable first patient group for such a trial.

### 4.3 Other pharmacological therapies

There are numerous suggestions and attempts of pharmacological therapy of sudden hearing loss based on the hypotheses of its etiology (an overview is found e.g. in [66]). In summary, there is no sufficient evidence supporting the routine application of antiviral, rheological, thrombolytic, vasodilatory, or antioxidant drugs. The same is true for vitamins, Gingko biloba, or alternative therapeutic methods [2], [29], [40].

## 5 Local (intratympanic) therapy

In the last two decades, intratympanic therapy of inner ear diseases was placed more and more in the focus of research interests, in particular after the pioneering study by Parnes and co-authors (1999) [93]. Reports about pre-clinical and clinical developments in the field of local and cell-based inner ear therapy are increasingly published every year. They concern the indications as well as pharmacological approaches and application technologies. The main indications refer to ISSHL, Menière’s disease, and tinnitus. But also further indications are in the focus of pre-clinical and clinical research [78], [94].

The advantages of local versus systemic drug application are

1. bypassing the blood-brain barrier;
2. achieving higher drug levels in the inner ear;
3. avoiding “first pass” effects;
4. reduction of undesired systemic effects, and
5. lower quantities of the drugs needed.

For local drug application to the inner ear, the advantages are seen especially for i) drugs with a low therapeutic range; ii) drugs with large first-pass effects; iii) drugs with relevant undesired effects outside the ear, and iv) expensive drugs. These aspects apply for example for neurotransmitters and neurotransmitter antagonists, peptides, viral and non-viral gene transfer, and cell-based therapies [94], [95]. 

### 5.1 Therapy of sudden hearing loss with corticosteroids

In the last years, nearly 200 articles with case reports, case series without control groups, retrospective cohort studies, and also about 20 randomized controlled trials (RCTs) on therapy of sudden hearing loss with intratympanic glucocorticoid application were published. Despite the large number of publications on this topic, only relatively uncertain conclusions regarding the efficacy of intratympanic corticosteroids for sudden hearing loss may be drawn on the basis of the current data. Even carefully described patient cohorts with improvements of hearing threshold do not allow conclusions regarding the efficacy without providing data from a control group that underwent either no or another treatment (e.g. [96], [97], [98], [99], [100]). 

The detailed analysis of the about 20 RCTs (which should be the gold standard for clinical research), reveals that the quality of most of these RTCs is disappointing and the risk of bias is high. 

Various systematic review articles and meta-analyses dealt with the different therapeutic strategies of intratympanic corticosteroid application for sudden hearing loss [101], [102], [103], [104], [105], [106], [107], [108]. Because of the above-mentioned low level of evidence, the different drugs (e.g. dexamethasone or methylprednisolone), and the different application protocols, the following conclusions should be considered with care until more and in particular higher-quality trials are available.

Regarding the primary therapy of sudden hearing loss, systemic (low-/medium-dose) and intratympanic glucocorticoid therapy seem to be equally effective (or equally ineffective) [101], [103], [107]. Studies comparing primary intratympanic with systemic high-dose corticosteroid therapy are not available.

In cases of insufficient recovery of hearing after systemic therapy, current data suggest that intratympanic glucocorticoid therapy as secondary treatment is associated with a significantly higher probability of an improved hearing threshold [101], [103], [105], [106].

Primary combined therapy (intratympanic and systemic therapy) seems to be superior to primary systemic glucocorticoid therapy. However, the effect is clearly lower than the one for intratympanic secondary (reserve) therapy and the risk of bias of those trials is very high [102]. In a meta-analysis for studies with this approach, also non-randomized trials were included in the evaluation [109], [110]. In one of the included retrospective cohort studies, for example, 59 out of 300 patients were selected for the historical control group, which shows the susceptibility for bias [109]. Data on the efficacy of primary combined therapy in contrast to primary systemic high-dose prednisolone therapy are not available.

**Figure 5:**
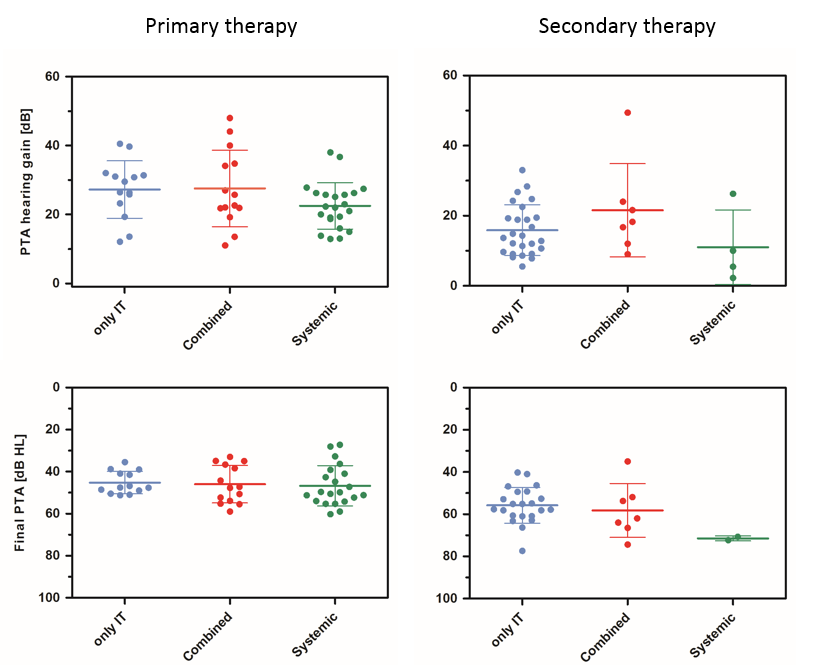
Comparison of the results of (randomized and non-randomized) trials on intratympanic, combined, and systemic primary and secondary therapy based on data from Liebau et al. [107], [111]. Left: Primary therapy of sudden hearing loss. Right: Secondary therapy (“salvage”, “rescue” therapy) after failed systemic therapy.

Summarizing the results of randomized as well as non-randomized trials and comparing intratympanic, systemic, and combined therapy, no significant difference was found between the different treatment strategies, neither regarding primary nor secondary therapy (Figure 5 (Fig. 5)). In addition, the tendency of a more relevant hearing improvement (not significant) in cases of early therapy onset with primary therapy of sudden hearing loss (intratympanic or combined) may be considered as a sham-effect, most likely being due to spontaneous recovery [107].

For secondary therapy, hearing improvement seems to be independent from treatment onset (2–4 weeks after sudden hearing loss or 4–6 weeks afterwards) [111]. 

**Figure 6:**
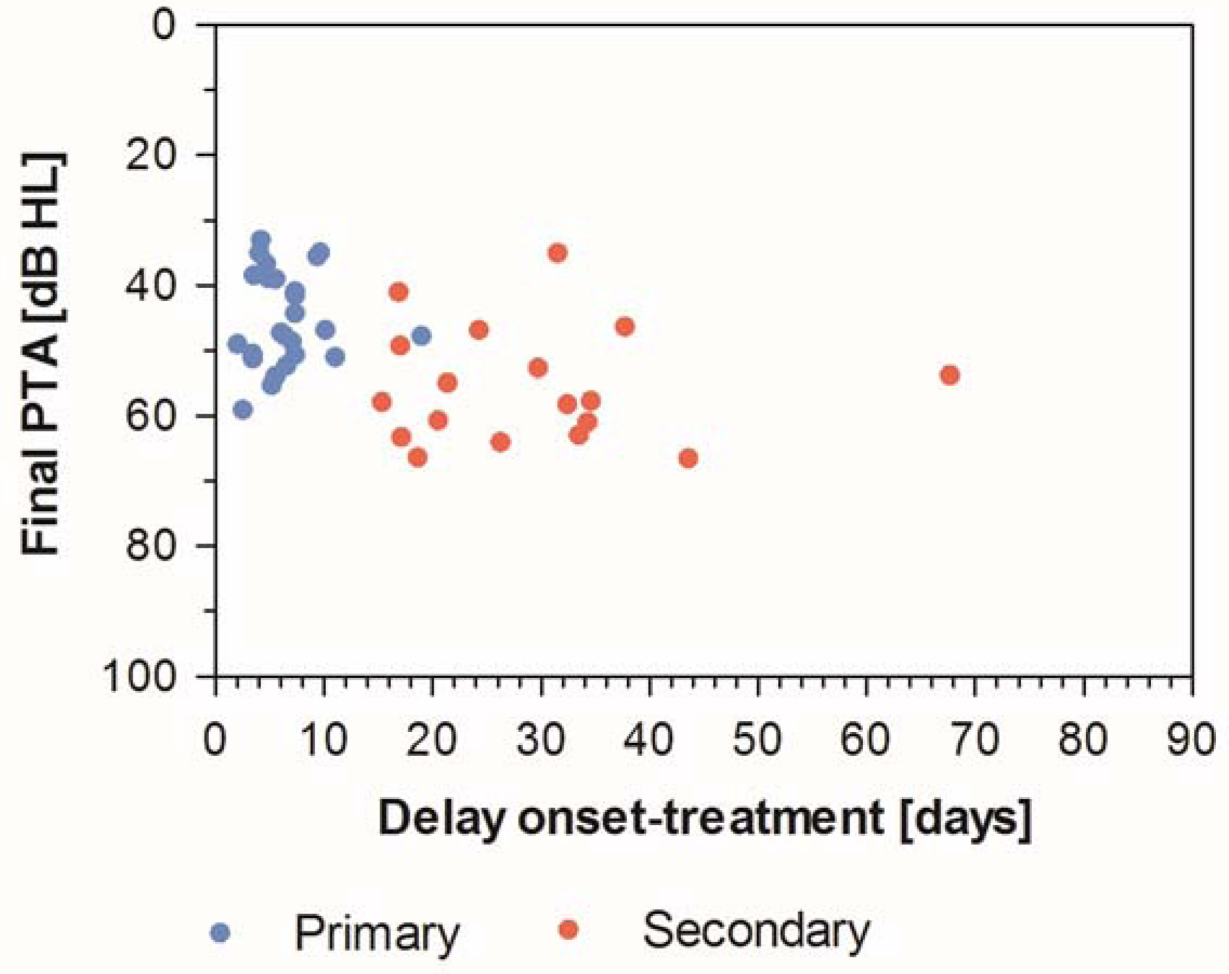
The finally achieved absolute hearing threshold seems to be independent from the onset of secondary therapy. This means that after 4–6 weeks after sudden hearing loss, intratympanic secondary therapy seems to lead to similar results compared to earlier (2–4 weeks) start of therapy.

If the final absolute hearing threshold is chosen as an outcome parameter – a parameter, which seems to be of higher importance for the patients than the change of hearing threshold, the relevance of which depends from the initial hearing loss – the final pure tone hearing threshold seems to be completely independent from the onset of treatment. This applies to primary as well as secondary therapy, at least for the available data with treatment onset within approximately 2 months after ISSHL (Figure 6 (Fig. 6)) [107], [111]. The meta-analyses revealed that the change of hearing threshold does not seem to be a suitable target parameter to assess the efficacy of the treatment of sudden hearing loss because this change depends from the initial hearing loss [107], [111]. 

Regarding primary intratympanic, primary combined, and secondary intratympanic therapy of sudden hearing loss with glucocorticoids, no correlation of individual parameters of the application protocol could be observed so far (type of drug [dexamethasone, methyl-/prednisolone], concentration, number of injections, interval of injection, total number of injections, total duration of therapy, duration of drug stay in the middle ear, time of end point measurement, patient’s age) [107], [111]. Missing correlations between dose and effect (Cmax; AUC) even raises the question if intratympanic glucocorticoid therapy is effective at all. 

**Figure 7:**
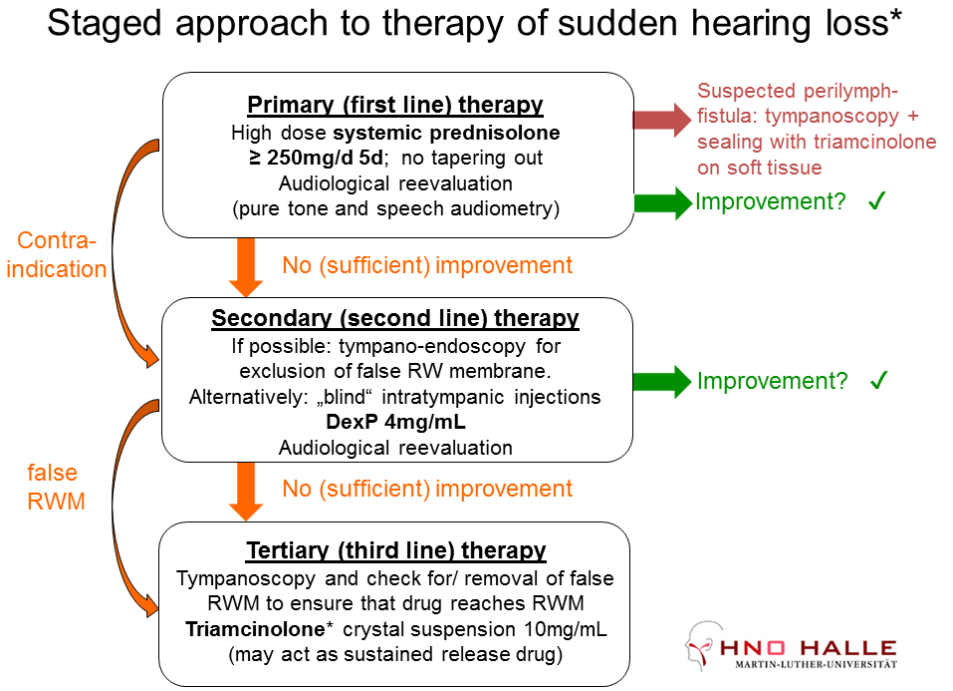
Staged approach to therapy of idiopathic sudden sensorinerual hearing loss (modified according to Plontke (2013) [7]). *as currently applied in the Department of Otorhinolaryngology, University Medicine Halle. RWM: round window membrane.

The staged approach of treating sudden hearing loss currently applied in the author’s department is presented in Figure 7 (Fig. 7). 

As primary therapy, systemic high-dose prednisolone administration is performed for 5 days (prednisolone i.v. 250 mg/d). Only in cases of contraindication for systemic high-dose prednisolone therapy (manifest psychosis, uncontrollable diabetes), a primary intratympanic therapy is performed. In very rare cases of suspected rupture of the round window membrane, e.g. in the context of barotrauma or mechanical trauma, tympanoscopy with exploration and sealing of the window is performed with triamcinolone acetonide soaked connective tissue.

As secondary therapy, “blind” (i.e. without inspection of the round window niche) transtympanic injection of dexamethasone-phosphate solution (sterile, pyrogen-free solution, about 0.3 ml, 4 mg/ml) is applied. After injection, the patients are asked to lay on the opposite side for 30 minutes.

**Figure 8:**
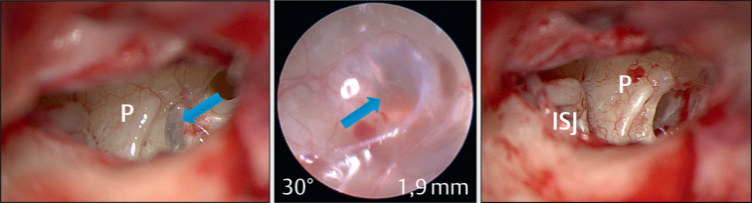
Obstruction of the round window niche with a “false” round window membrane (left). Endoscopic view (middle) and condition after removal of the false membrane (right). P: promontory; ISJ: incudo-stapedial joint.

**Figure 9:**
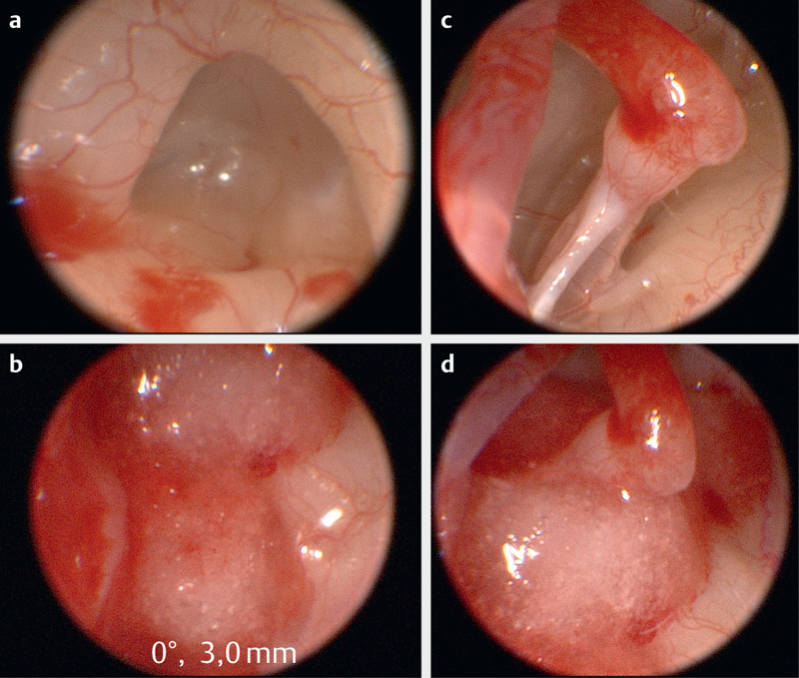
Tertiary therapy of sudden hearing loss with tympanoscopy and application of triamcinolone 10 mg/ml on Curaspon^®^ into the oval (c, d) and round window niche (a, b).

Tertiary therapy consists of tympanoscopy and inspection of the middle ear to exclude or remove so-called “false round window membranes” that obstruct the round window niche in about 25–33% of the cases [38], [39], [112] (Figure 8 (Fig. 8)). In order to achieve a depot effect, Curaspon^®^ sponges soaked with triamcinolone acetonide are inserted into the niches of the round and oval windows (Figure 9 (Fig. 9)).

### 5.2 Recent developments

Because of the high prevalence of inner ear diseases, “small” and large pharmaceutical companies are interested in this topic. Beside start-up companies that exclusively dedicate their work to this subject, even large pharmaceutical companies meanwhile invest in this field [113]. The current developments consider drugs and drug application systems alike [95], [114], [115], [116]. 

**Figure 10:**
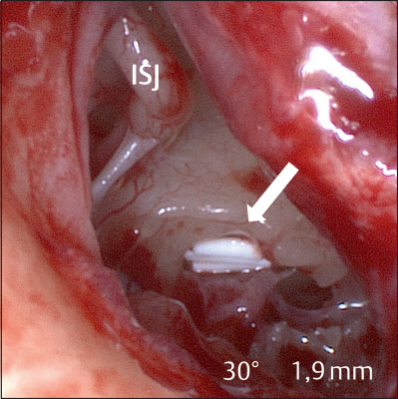
Bioabsorbable drug carrier (OZURDEX^®^ arrow) in the round window niche for continuous release of dexamethasone [117]. Endoscopic view into the right middle ear. ISJ: incudo-stapedial joint.

With respect to drug application systems, biocompatible, absorbable polymers (as gels or solids) will probably play a major role in the future. They allow targeted and controlled release of substances over a predefined time period outside or inside the cochlea (Figure 10 (Fig. 10)) [95], [115], [116], [117], [118].

Considering drug development, AM111, an antiapoptotic peptide, that needs to be injected intratympanically as early as possible after acute acoustic trauma or sudden hearing loss is currently under clinical investigation. First studies demonstrated the safety of the procedure [119], [120]. A placebo-controlled study examined the efficacy of two different dosages of AM111. In the target study population, there was no difference in the hearing improvement between the groups. In an explorative, hypothesis-generating (post-hoc) subgroup analysis, patients with severe hearing losses in the group receiving low dose AM111 revealed significant differences (favoring AM111) on day 7 and day 30, but not after 90 days and not in the group receiving the higher-dose of the test substance [120]. 

## 6 Need for research and development

### 6.1 Necessity of clinical trials

In preclinical and clinical research – due to different causes - an increasing number of biomedical journals and publications contrasts with a decreasing percentage of high-quality and biometrically sufficiently supported clinical data regarding study design, performance, and reporting. Initially promising ideas and hypotheses often do not lead to expected improvements in healthcare [121]. Chalmers and Glasziou identified the reasons for avoidable waste in biomedical research [122]. They assume that even without considering (possible) inefficiency in regulation and research management, about 85% of the investments in biomedical research are a waste of efforts. Detailed solutions were published in a series of articles in the journal *The Lancet* (“The Lancet Research: Increasing Value, Reducing Waste Series” [121]).

### 6.2 HODOKORT trial

**Figure 11:**
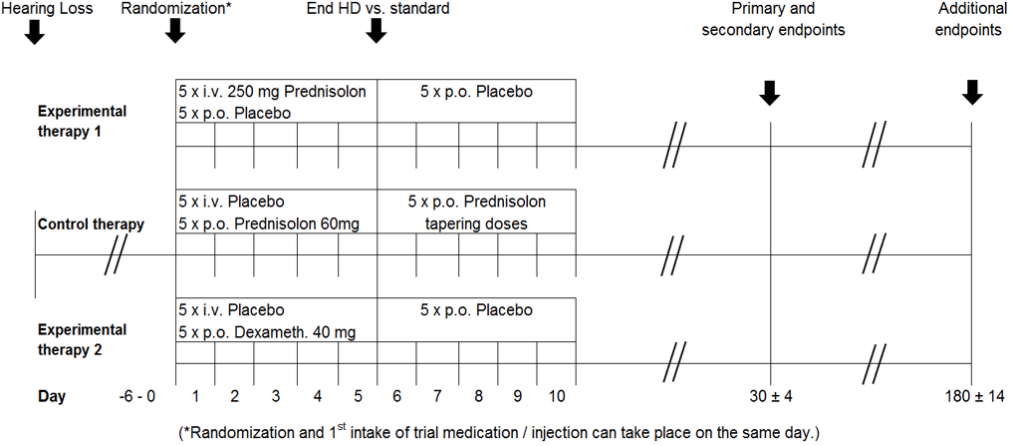
HODOKORT study (http://hodokort-studie.hno.org/) [86]: The triple-blind, three-armed study with parallel group design encompasses two different high-dose corticosteroid therapies (intravenous prednisolone or oral dexamethasone application in an equivalent dosage) as well as a control group (middle line) receiving the internationally recommended lower-dose standard therapy. German Study Center for Otolaryngology, Head & Neck Surgery (DSZ-HNO) of the German Society of Otolaryngology, Head & Neck Surgery and the German Association of Otolaryngologists; Principle investigator (PI): Stefan K Plontke (Halle/Saale); Study Coordination: Coordination Center for Clinical Trials of Halle, University Medicine Halle, Martin Luther University Halle-Wittenberg; sponsored by the Research Funding Program on “Clinical Studies with high Relevance for Patient Care” in the context of health research program of the Federal Ministry for Research and Development (BMBF), Germany. The figure is taken from the study protocol©, courtesy of Martin Luther University Halle-Wittenberg.

Based on the insufficient evidence for efficacy of systemic high-dose glucocorticoid therapy for sudden hearing loss, a current multicenter national randomized clinical trial aims at determining the efficacy of intravenous or oral primary systemic high-dose glucocorticoid therapy compared to the internationally recommended standard dose therapy for the treatment of unilateral acute idiopathic sudden hearing loss (http://hodokort-studie.hno.org/) (Figure 11 (Fig. 11)). This trial is fully sponsored by the program of healthcare research of the Federal Ministry of Research and Development (BMBF) and is one of the first two trials of the German Study Center of Otolaryngology, Head & Neck Surgery. The German Study Center of Otolaryngology, Head & Neck Surgery (DSZ-HNO) is a cooperation project of the German Society of Otolaryngology, Head & Neck Surgery, the German Association of Otolaryngologists, the German Registry of Clinical Studies, and the Study Center of the University Hospitals of Freiburg, Germany [86].

### 6.3 Definitions, inclusion criteria, and outcome parameters for studies regarding sudden hearing loss

Up to now there is no internationally standardized definition regarding audiological parameters of sudden hearing loss. The frequently mentioned “30 dB in 3 subsequent frequencies” does not define a frequency range for this hearing loss [29]. Many trials do not even include this criterion of 3x30 dB [66]. Average values of the pure tone hearing threshold over different frequencies are taken and the calculation of sudden hearing loss and improvement is performed in very different ways [123].

Most of the studies refer to an absolute hearing threshold as inclusion criterion and as disease-related hearing loss. In the HODOKORT trial the current pure tone audiogram is either compared with a previous audiogram of the affected side (if available), the audiogram of the contralateral side, or the age- and gender-specific normal audiogram (DIN ISO 7029). As frequency range for the main outcome parameter, the 3 frequencies are considered that are most affected by the hearing loss, as already suggested in other studies [120], [124]. For calculation of the disease-related hearing loss, a free (open source) Microsoft EXCEL based software tool is available, that can also be used as screening macro for checking the inclusion criteria for clinical trials [125].

Another problem of clinical studies on sudden hearing loss is that for mild and moderate hearing losses, improvements of hearing threshold achieved with or without treatment cannot be statistically differentiated. Hence, only the inclusion of patients with a certain minimum hearing loss is currently recommended for clinical studies on ISSHL [86], [120], [126], [127].

Up to now, there is no international consensus in terms of measuring treatment success. The pure tone hearing threshold, however, is currently the internationally most standardized parameter. In most of the trials on sudden hearing loss or other inner ear diseases, the change of hearing threshold is reported. Even current high-quality randomized clinical trials on sudden hearing loss apply this parameter. Speech understanding (percentage of the correctly understood monosyllables of the Freiburg test at 65 and 80 dB SPL), but especially speech understanding in noise even better reflect how a patient may communicate in daily life. Nevertheless, it is not easy to compare the results of speech audiometries of different languages. In addition, they are influenced by other parameters such as speech competence and cognitive factors.

There are different possibilities to classify improvement of hearing based on the changes of pure tone or speech reception threshold. Internationally, however, no consensus could be found on when hearing improvement can be considered as helpful or meaningful. For example, hearing aids are used for a broad range of hearing thresholds. Furthermore, a current American guideline emphasized that defined grades of improvement may be associated with different levels of benefit for the individual patient [29].

Specific patient-related and patient reported outcome parameters on the quality of life such as for example the patient’s self-assessment of the general health-related quality of life (score of physical and psychical summation scale in the SF12 questionnaire) and the assessment of the subjective hearing handicap (score of the HHIE questionnaire) or other standardized and validated QoL instruments should be involved more frequently in the future.

## 7 Conclusion

Even from a prognostic point of view, sudden hearing loss is not an emergency that has to be treated immediately [2]. This relates to sudden idiopathic hearing loss, i.e. hearing loss of unknown etiology. Severe or even life-threatening diseases of which acute sudden hearing loss is only a symptom, have to be excluded. Because of numerous possible differential diagnoses of acute sensorineural hearing losses, this symptom should lead to targeted, history-related **diagnostics**. Patients with acute unilateral cochlea-vestibular symptoms should undergo appropriate MRI examination (thin slices with contrast medium, which represent the current gold standard) to assess peripheral (inner ear) and retro-cochlear/central causes according to the recommendations of the German Radiological Society (http://www.ag-kopf-hals.drg.de/de-DE/295/stellungnahmen-und-empfehlungen). Requesting an MRI examination to clarify the causes of sudden hearing loss, it is recommened to ask explicitly for the exclusion of an intralabyrinthine schwannoma. New developments of imaging diagnostics improve the possibilities of differential diagnostics, e.g. the diagnosis of endolymphatic hydrops.

The study situation with respect to the **therapy** of sudden hearing loss is very unsatisfactory – regarding the quality, not the quantity of trials. There is an existing rational basis for the treatment of acute cochlea-vestibular disorders with corticosteroids. The value of systemically applied corticosteroids in the initial treatment of sudden hearing loss (international standard with low/ medium systemic doses) remains unclear. Regarding **primary therapy** of sudden hearing loss, **systemic corticosteroids** (low/medium dose) and **intratympanic corticosteroids** seem to be equally effective (or equally ineffective). Up to now there is no sufficient evidence for the efficacy of primary **systemic high-dose glucocorticoid therapy** for sudden hearing loss as recommended in the current German AWMF guideline. To answer this question, a multicenter clinical trial entitled “Efficacy and safety of high dose glucocorticosteroid treatment for idiopathic sudden sensorineural hearing loss – a three-armed, randomized, triple-blind, multicenter trial (**HODOKORT**)” is performed supported by the German Study Center of Otolaryngology, Head & Neck Surgery (http://hodokort-studie.hno.org/). 

**Primary combined therapy** (intratympanic and low-/medium dose systemic application) seems to be superior to primary low-dose systemic glucocorticoid therapy. However, the effect is clearly lower compared to intratympanic secondary (reserve) therapy (see below) and the risk of bias in these trials is very high. Data on the efficacy of a primary combined therapy compared to primary systemic high-dose prednisolone therapy are not available.

The tendency of increased hearing improvement after early onset of primary therapy of sudden hearing loss (intratympanic or combined) can be considered a sham effect because this is most likely to be due to spontaneous recovery.

In case of insufficient recovery of hearing after systemic therapy of sudden hearing loss, current data suggest that intratympanic glucocorticoid therapy as **secondary therapy** is associated with a significantly higher probability of an improved hearing. Hereby, the hearing improvement seems to be independent from the onset of therapy (2–4 weeks after sudden hearing loss or 4–6 weeks afterwards). Therefore, the necessity of early onset of intratympanic secondary therapy is not supported on the basis of the currently available data. 

In the context of primary intratympanic, primary combined (intratympanic and internationally accepted systemic standard therapy), and secondary intratympanic therapy of sudden hearing loss with glucocorticoids, the therapeutic success does not seem to depend on individual parameters of the application protocol (type of substance [dexamethasone, methyl-prednisolone], concentration, number of injections, interval of injections, total number of injections, duration of injection, time of the drug to remains in the middle ear, time of endpoint measuring, patient’s age) so that currently no recommendation for a specific therapy protocol of intratympanic therapy can be given.

## Notes

### Acknowledgements

This work was partially done within the project KS2013-190 supported by the Federal Ministry of Education and Science in Germany (BMBF-Programm „Klinische Studien mit hoher Relevanz für die Patientenversorgung“ im Rahmenprogramm Gesundheitsforschung. I want to thank Prof. Gerhard Hesse, Prof. Robert Gürkov, Prof. Thorsten Rahne, Dr. Arne Liebau, and Prof. Dirk Eßer for comments on the original German version of this manuscript. Further, I thank Prof. Rahne for his contributions to the chapter on “Auditory evoked brainstem potentials” and his support regarding the figures 1–3 and Dr. Liebau for creating the figures 5 and 6. I thank Mrs. Susanne Zapf, Marburg, and the German Society of Oto-Rhino-Laryngology, Head and Neck Surgery for the translation of the German version of the manuscript into English.

### Competing interests

The author is or has been a consultant to companies with questions regarding inner ear physiology and pathophysiology, inner ear diseases, and therapy of inner ear disorders including trial design, drugs and drug applications strategies (Otonomy Inc., San Diego USA; Hoffmann-La Roche, Basel, Switzerland; Boehringer Ingelheim Pharma GmbH & Co. KG; Ingelheim am Rhein, Germany). The author is head of the scientific advisory board of AudioCure Pharma GmbH, Berlin, Germany. The authors institution receives research grant support from MedEl, Austria and MedEl, Germany. The author received honorary for lectures in this topic from the ENT-Physician’s organisation in Germany and Infectopharm, Heppenheim, Germany. The author received travel support, e.g. for lectures from Cochlear Deutschland GmbH & Co. KG. The author received a major research grant from the Federal Ministry of Education and Science in Germany (BMBF: KS2013-190). The author also received honorary for lectures or session moderations not related to this topic by Merck Serono, Darmstadt, Germany.

## Figures and Tables

**Table 1:**
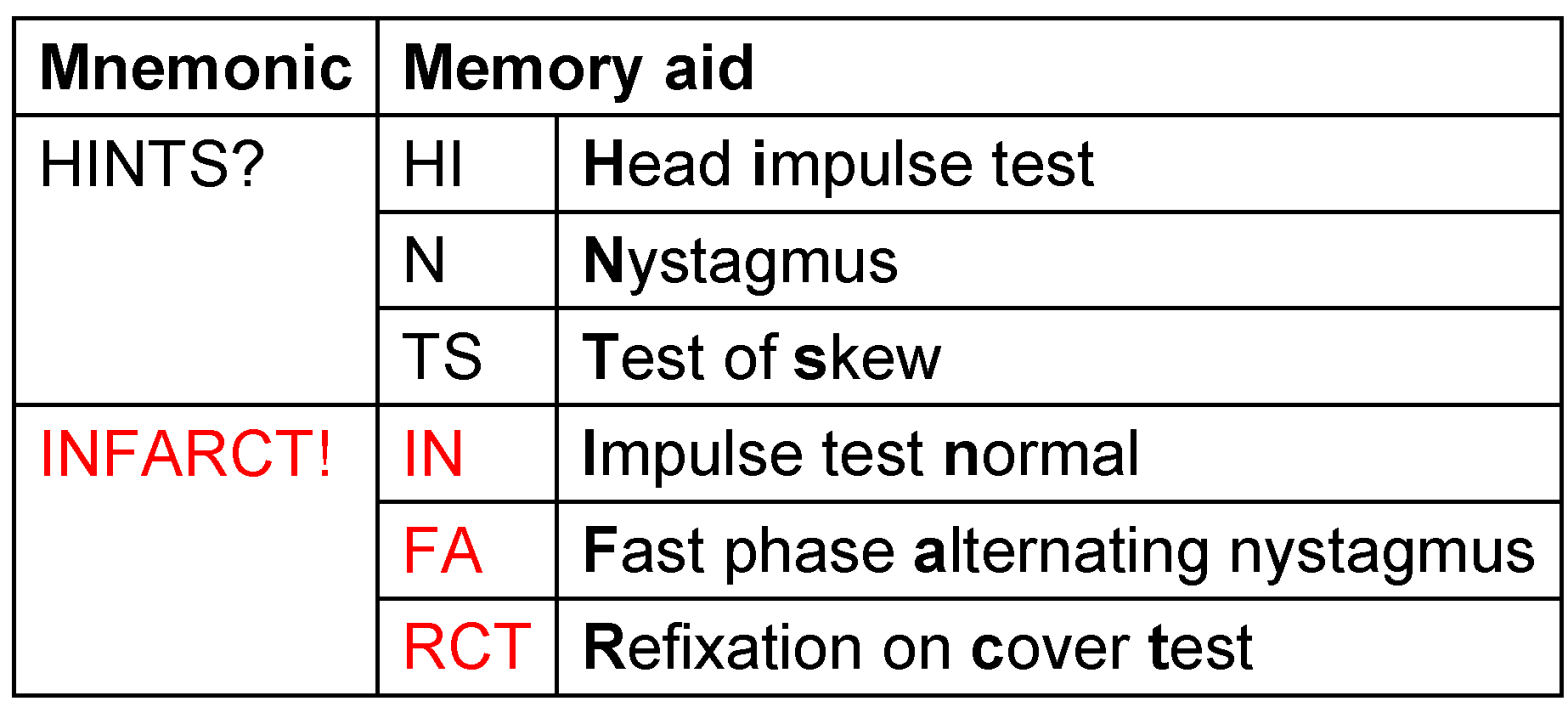
HINTS for diagnosing stroke in acute vestibular syndrome (adapted from Kattah et al. [10)]

**Table 2:**
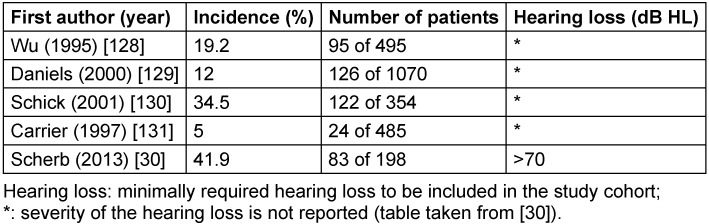
Percentages of pathological MRI findings in patients with sudden hearing loss (selected articles)

**Table 3:**
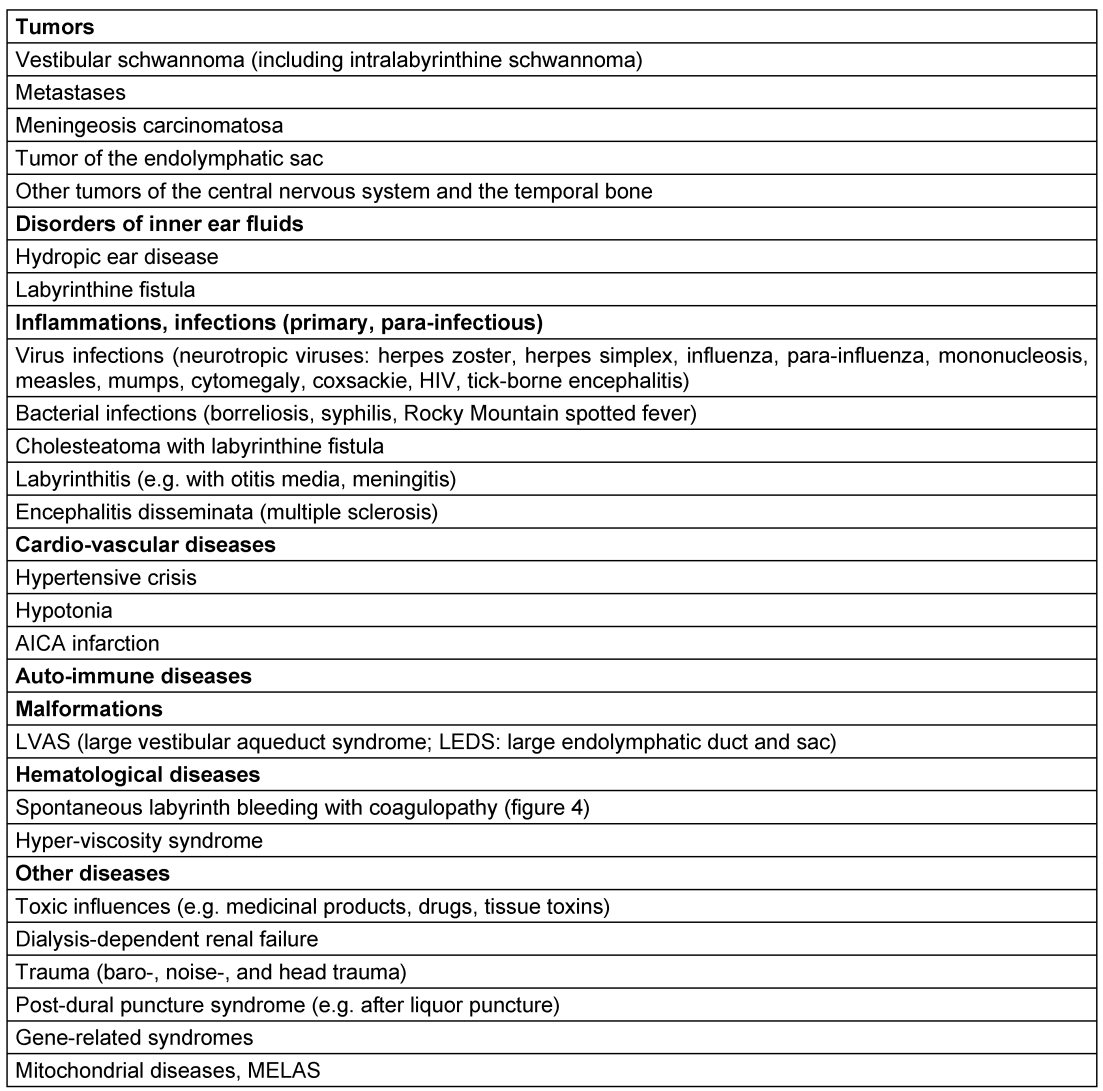
Differential diagnostics of sudden hearing loss (modified according to [2], [6], [7], [29], [40])

**Table 4:**
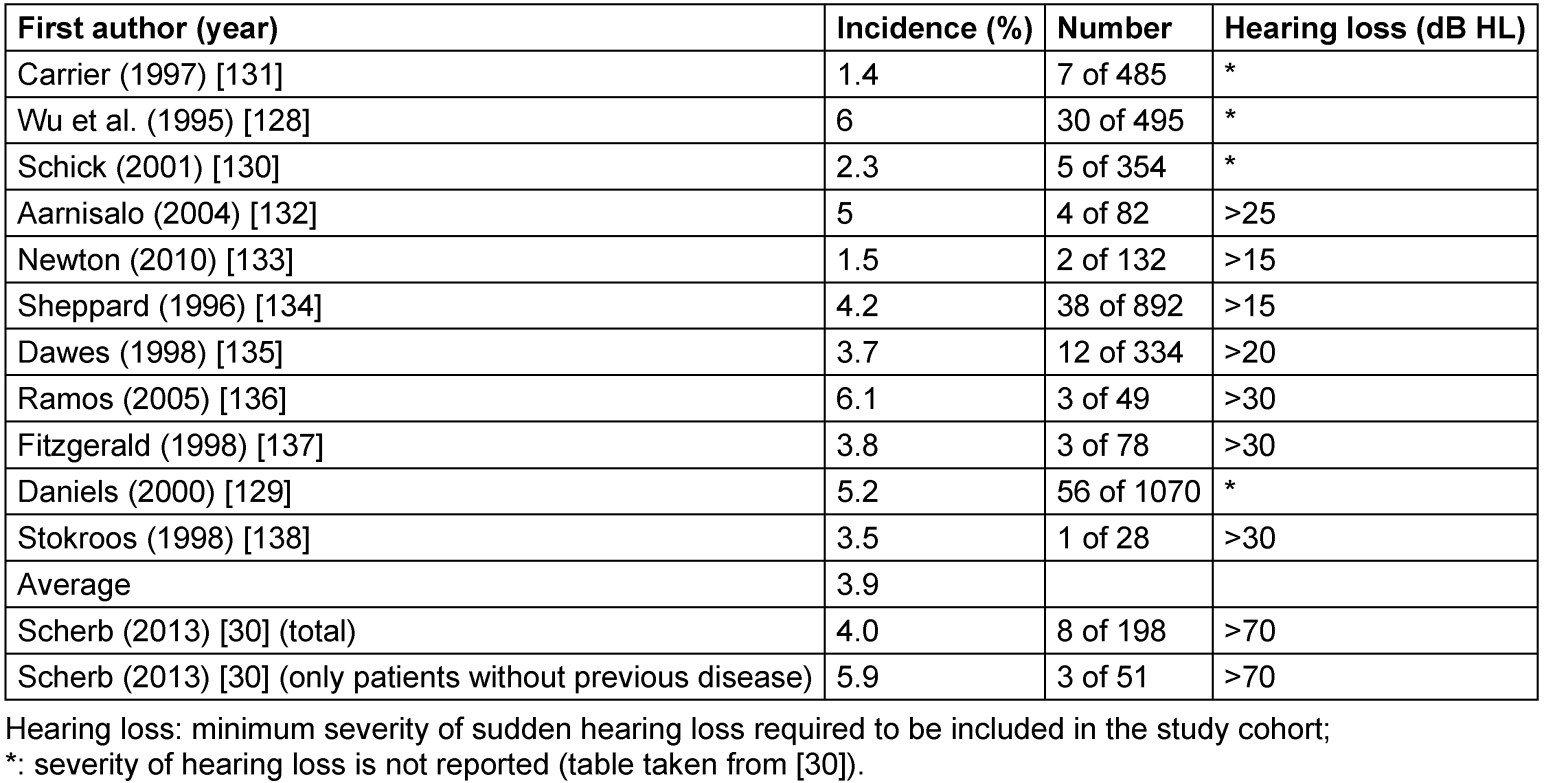
Vestibular schwannoma in studies on MRI diagnostics in the context of sudden hearing loss

## References

[1] von Gablenz P, Holube I (2015). Prävalenz von Schwerhörigkeit im Nordwesten Deutschlands : Ergebnisse einer epidemiologischen Untersuchung zum Hörstatus (HÖRSTAT). HNO.

[2] Deutsche Gesellschaft für Hals-Nasen-Ohren-Heilkunde, Kopf- und Hals-Chirurgie (2014). Leitlinie Hörsturz (Akuter idiopathischer sensorineuraler Hörverlust).

[3] Byl FM (1984). Sudden hearing loss: eight years' experience and suggested prognostic table. Laryngoscope.

[4] Olzowy B, Osterkorn D, Suckfüll M (2005). Praktische Erfahrung bestätigt. Hörsturz wesentlich häufiger als bisher angenommen. MMW Fortschr Med.

[5] Klemm E, Deutscher A, Mösges R (2009). Aktuelle Stichprobe zur Epidemiologie des idiopathischen Hörsturzes. Laryngorhinootologie.

[6] Plontke S, Reiß M (2009). Schwerhörigkeit, Taubheit, Tinnitus. Facharztwissen HNO-Heilkunde, Differenzierte Diagnostik und Therapie.

[7] Plontke S (2013). Notfälle der Sinnesorgane im HNO-Gebiet. Laryngorhinootologie.

[8] Plontke SK, Walther LE (2014). Differenzialdiagnose „Schwindel“. Laryngorhinootologie.

[9] Volgger V, Krause E, Ertl-Wagner B, Gürkov R (2016). Hydropische Ohrerkrankung vom vestibulären Typ. HNO.

[10] Kattah JC, Talkad AV, Wang DZ, Hsieh YH, Newman-Toker DE (2009). HINTS to diagnose stroke in the acute vestibular syndrome: three-step bedside oculomotor examination more sensitive than early MRI diffusion-weighted imaging. Stroke.

[11] Brandt T, Dieterich M, Strupp M (2013). Vertigo-Leitsymptom Schwindel.

[12] Zelle D, Dalhoff E, Gummer AW (2016). Objektive Hördiagnostik mit DPOAE : Neue Erkenntnisse zur Generierung und klinischen Anwendung. HNO.

[13] Chao TK, Chen TH (2006). Distortion product otoacoustic emissions as a prognostic factor for idiopathic sudden sensorineural hearing loss. Audiol Neurootol.

[14] Hoth S (2005). On a possible prognostic value of otoacoustic emissions: a study on patients with sudden hearing loss. Eur Arch Otorhinolaryngol.

[15] Mori T, Suzuki H, Hiraki N, Hashida K, Ohbuchi T, Katoh A, Udaka T (2011). Prediction of hearing outcomes by distortion product otoacoustic emissions in patients with idiopathic sudden sensorineural hearing loss. Auris Nasus Larynx.

[16] Hoth S (2016). Der Freiburger Sprachtest : Eine Säule der Sprachaudiometrie im deutschsprachigen Raum. HNO.

[17] Schmidt T, Baljić I (2016). Untersuchung zum Trainingseffekt des Freiburger Einsilbertests. HNO.

[18] Hoppe U (2016). Hörgeräteerfolgskontrolle mit dem Freiburger Einsilbertest. HNO.

[19] Müller A, Hocke T, Hoppe U, Mir-Salim P (2016). Der Einfluss des Alters bei der Evaluierung des funktionellen Hörgerätenutzens mittels Sprachaudiometrie. HNO.

[20] Thümmler R, Liebscher T, Hoppe U (2016). Einfluss einer Hörgeräteversorgung auf das Einsilberverstehen und das subjektiv erlebte Alltagshören. HNO.

[21] Barrs DM, Brackmann DE, Olson JE, House WF (1985). Changing concepts of acoustic neuroma diagnosis. Arch Otolaryngol.

[22] Selters WA, Brackmann DE (1977). Acoustic tumor detection with brain stem electric response audiometry. Arch Otolaryngol.

[23] Gentry LR, Jacoby CG, Turski PA, Houston LW, Strother CM, Sackett JF (1987). Cerebellopontine angle-petromastoid mass lesions: comparative study of diagnosis with MR imaging and CT. Radiology.

[24] Naessens B, Gordts F, Clement PA, Buisseret T (1996). Re-evaluation of the ABR in the diagnosis of CPA tumors in the MRI-era. Acta Otorhinolaryngol Belg.

[25] Koors PD, Thacker LR, Coelho DH (2013). ABR in the diagnosis of vestibular schwannomas: a meta-analysis. Am J Otolaryngol.

[26] Berenholz LP, Eriksen C, Hirsh FA (1992). Recovery from repeated sudden hearing loss with corticosteroid use in the presence of an acoustic neuroma. Ann Otol Rhinol Laryngol.

[27] Nageris BI, Popovtzer A (2003). Acoustic neuroma in patients with completely resolved sudden hearing loss. Ann Otol Rhinol Laryngol.

[28] Baljić I, Börner-Lünser E, Eßer D, Guntinas-Lichius O (2017). Frühe akustisch evozierte Potenziale bei Patienten mit „kleinem“ Vestibularisschwannom. HNO.

[29] Stachler RJ, Chandrasekhar SS, Archer SM, Rosenfeld RM, Schwartz SR, Barrs DM, Brown SR, Fife TD, Ford P, Ganiats TG, Hollingsworth DB, Lewandowski CA, Montano JJ, Saunders JE, Tucci DL, Valente M, Warren BE, Yaremchuk KL, Robertson PJ, American Academy of Otolaryngology-Head and Neck Surgery (2012). Clinical practice guideline: sudden hearing loss. Otolaryngol Head Neck Surg.

[30] Scherb R (2013). Wertigkeit der MRT zur Abklärung des hochgradigen Hörsturzes [Inaugural-Dissertation zur Erlangung des Doktorgrades der Medizin].

[31] Bartel-Friedrich S, Amaya B, Rasinski C, Fuchs M, Kösling S (2008). Der erweiterte Ductus und Saccus endolymphaticus : Teil 1: Analyse bildgebender Befunde. HNO.

[32] Koesling S, Rasinski C, Amaya B (2006). Imaging and clinical findings in large endolymphatic duct and sac syndrome. Eur J Radiol.

[33] Gedlicka C, Formanek M, Ehrenberger K (2009). Analysis of 60 patients after tympanotomy and sealing of the round window membrane after acute unilateral sensorineural hearing loss. Am J Otolaryngol.

[34] Haubner F, Rohrmeier C, Koch C, Vielsmeier V, Strutz J, Kleinjung T (2012). Occurence of a round window membrane rupture in patients with sudden sensorineural hearing loss. BMC Ear Nose Throat Disord.

[35] Kampfner D, Anagiotos A, Luers JC, Hüttenbrink KB, Preuss SF (2014). Analysis of 101 patients with severe to profound sudden unilateral hearing loss treated with explorative tympanotomy and sealing of the round window membrane. Eur Arch Otorhinolaryngol.

[36] Loader B, Atteneder C, Kaider A, Franz P (2013). Tympanotomy with sealing of the round window as surgical salvage option in sudden idiopathic sensorineural hearing loss. Acta Otolaryngol.

[37] Reineke U, Hühnerschulte M, Ebmeyer J, Sudhoff H (2013). Tympanoskopie mit Abschottung der Rundfenstermembran beim idiopathischen Hörsturz: Eine retrospektive Analyse. HNO.

[38] Alzamil KS, Linthicum FH (2000). Extraneous round window membranes and plugs: possible effect on intratympanic therapy. Ann Otol Rhinol Laryngol.

[39] Plontke SK (2011). Evaluation of the round window niche before local drug delivery to the inner ear using a new mini-otoscope. Otol Neurotol.

[40] Arts AH, Flint WP, Haughey BH, Lund VJ, Niparko JK, Richardson MA, Robbins KT, Thomas JR (2010). Sensorineural Hearing Loss. Cummings Otolaryngology Head and Neck Surgery 3.

[41] Hoch S, Vomhof T, Teymoortash A (2015). Critical evaluation of round window membrane sealing in the treatment of idiopathic sudden unilateral hearing loss. Clin Exp Otorhinolaryngol.

[42] Sara SA, Teh BM, Friedland P (2014). Bilateral sudden sensorineural hearing loss: review. J Laryngol Otol.

[43] Gluth MB, Baratz KH, Matteson EL, Driscoll CL (2006). Cogan syndrome: a retrospective review of 60 patients throughout a half century. Mayo Clin Proc.

[44] Mösges R, Köberlein J, Heibges A, Erdtracht B, Klingel R, Lehmacher W, RHEO-ISHL Study Group (2009). Rheopheresis for idiopathic sudden hearing loss: results from a large prospective, multicenter, randomized, controlled clinical trial. Eur Arch Otorhinolaryngol.

[45] Teranishi M, Naganawa S, Katayama N, Sugiura M, Nakata S, Sone M, Nakashima T (2009). Image evaluation of endolymphatic space in fluctuating hearing loss without vertigo. Eur Arch Otorhinolaryngol.

[46] Gürkov R, Pyykö I, Zou J, Kentala E (2016). What is Menière's disease? A contemporary re-evaluation of endolymphatic hydrops. J Neurol.

[47] Dubernard X, Somers T, Veros K, Vincent C, Franco-Vidal V, Deguine O, Bordure P, Linder T, Lescanne E, Ayache D, Mondain M, Schmerber S, Dahmani-Causse M, Truy E, Darrouzet V (2014). Clinical presentation of intralabyrinthine schwannomas: a multicenter study of 110 cases. Otol Neurotol.

[48] Jerin C, Krause E, Ertl-Wagner B, Gürkov R (2016). Klinische Eigenschaften von „delayed endolymphatic hydrops“ und intralabyrinthärem Schwannom : Eine bildgebungsbasierte komparative Fallserie. HNO.

[49] Just T, Hingst V, Pau HW (2010). Intracochleäres Schwannom: Eine Differenzialdiagnose bei menièriformen Beschwerden. Laryngorhinootologie.

[50] Kennedy RJ, Shelton C, Salzman KL, Davidson HC, Harnsberger HR (2004). Intralabyrinthine schwannomas: diagnosis, management, and a new classification system. Otol Neurotol.

[51] Salzman KL, Childs AM, Davidson HC, Kennedy RJ, Shelton C, Harnsberger HR (2012). Intralabyrinthine schwannomas: imaging diagnosis and classification. AJNR Am J Neuroradiol.

[52] Van Abel KM, Carlson ML, Link MJ, Neff BA, Beatty CW, Lohse CM, Eckel LJ, Lane JI, Driscoll CL (2013). Primary inner ear schwannomas: a case series and systematic review of the literature. Laryngoscope.

[53] Plontke SK, Kösling S, Pazaitis N, Rahne T (2017). Intracochlear schwannoma : Tumor removal via subtotal cochleoectomy and partial cochlear reconstruction with preservation of semicircular canal function. HNO.

[54] Kösling S (2011). Intralabyrinthäre Schwannome aus radiologischer Sicht. HNO.

[55] Slattery EL, Babu SC, Chole RA, Zappia JJ (2015). Intralabyrinthine schwannomas mimic cochleovestibular disease: symptoms from tumor mass effect in the labyrinth. Otol Neurotol.

[56] Tieleman A, Casselman JW, Somers T, Delanote J, Kuhweide R, Ghekiere J, De Foer B, Offeciers EF (2008). Imaging of intralabyrinthine schwannomas: a retrospective study of 52 cases with emphasis on lesion growth. AJNR Am J Neuroradiol.

[57] Aschendorff A, Arndt S, Laszig R, Wesarg T, Hassepaß F, Beck R (2017). Therapie und Hörrehabilitation intralabyrinthärer Schwannome mittels Cochlear Implant : Englische Version Treatment and auditory rehabilitation of intralabyrinthine schwannoma by means of cochlear implants : English version. HNO.

[58] Plontke SK, Rahne T, Pfister M, Götze G, Heider C, Pazaitis N, Strauss C, Caye-Thomasen P, Kösling S (2017). Intralabyrinthine schwannomas : Surgical management and hearing rehabilitation with cochlear implants. HNO.

[59] Bartel-Friedrich S, Fuchs M, Amaya B, Rasinski C, Meuret S, Kösling S (2008). Der erweiterte Ductus und Saccus endolymphaticus : Teil 2: Klinische Manifestationen. HNO.

[60] Nordström CK, Laurell G, Rask-Andersen H (2016). The Human Vestibular Aqueduct: Anatomical Characteristics and Enlargement Criteria. Otol Neurotol.

[61] Wick CC, Manzoor NF, Semaan MT, Megerian CA (2015). Endolymphatic sac tumors. Otolaryngol Clin North Am.

[62] Naganawa S, Nakashima T (2014). Visualization of endolymphatic hydrops with MR imaging in patients with Ménière's disease and related pathologies: current status of its methods and clinical significance. Jpn J Radiol.

[63] Baráth K, Schuknecht B, Naldi AM, Schrepfer T, Bockisch CJ, Hegemann SC (2014). Detection and grading of endolymphatic hydrops in Menière disease using MR imaging. AJNR Am J Neuroradiol.

[64] Martines F, Dispenza F, Gagliardo C, Martines E, Bentivegna D (2011). Sudden sensorineural hearing loss as prodromal symptom of anterior inferior cerebellar artery infarction. ORL J Otorhinolaryngol Relat Spec.

[65] Lee H (2012). Audiovestibular loss in anterior inferior cerebellar artery territory infarction: a window to early detection?. J Neurol Sci.

[66] Plontke S (2005). Therapy of hearing disorders - conservative procedures. GMS Curr Top Otorhinolaryngol Head Neck Surg.

[67] Trune DR, Canlon B (2012). Corticosteroid therapy for hearing and balance disorders. Anat Rec (Hoboken).

[68] Müller M, Tisch M, Maier H, Löwenheim H (2016). Begrenzung chronischer Hörverluste durch lokale Glukokortikoidgabe : Meerschweinchen mit akutem Lärmtrauma. HNO.

[69] Sendowski I, Abaamrane L, Raffin F, Cros A, Clarençon D (2006). Therapeutic efficacy of intra-cochlear administration of methylprednisolone after acoustic trauma caused by gunshot noise in guinea pigs. Hear Res.

[70] Takemura K, Komeda M, Yagi M, Himeno C, Izumikawa M, Doi T, Kuriyama H, Miller JM, Yamashita T (2004). Direct inner ear infusion of dexamethasone attenuates noise-induced trauma in guinea pig. Hear Res.

[71] Wang Y, Hirose K, Liberman MC (2002). Dynamics of noise-induced cellular injury and repair in the mouse cochlea. J Assoc Res Otolaryngol.

[72] Harrop-Jones A, Wang X, Fernandez R, Dellamary L, Ryan AF, LeBel C, Piu F (2016). The Sustained-Exposure Dexamethasone Formulation OTO-104 Offers Effective Protection against Noise-Induced Hearing Loss. Audiol Neurootol.

[73] Himeno C, Komeda M, Izumikawa M, Takemura K, Yagi M, Weiping Y, Doi T, Kuriyama H, Miller JM, Yamashita T (2002). Intra-cochlear administration of dexamethasone attenuates aminoglycoside ototoxicity in the guinea pig. Hear Res.

[74] Fernandez R, Harrop-Jones A, Wang X, Dellamary L, LeBel C, Piu F (2016). The Sustained-Exposure Dexamethasone Formulation OTO-104 Offers Effective Protection against Cisplatin-Induced Hearing Loss. Audiol Neurootol.

[75] Kim HH, Addison J, Suh E, Trune DR, Richter CP (2007). Otoprotective effects of dexamethasone in the management of pneumococcal meningitis: an animal study. Laryngoscope.

[76] Trune DR, Kempton JB (2010). Low dose combination steroids control autoimmune mouse hearing loss. J Neuroimmunol.

[77] Trune DR, Kempton JB, Gross ND (2006). Mineralocorticoid receptor mediates glucocorticoid treatment effects in the autoimmune mouse ear. Hear Res.

[78] Plontke SK, Götze G, Rahne T, Liebau A (2017). Intracochlear drug delivery in combination with cochlear implants : Current aspects. HNO.

[79] Conlin AE, Parnes LS (2007). Treatment of sudden sensorineural hearing loss: II. A Meta-analysis. Arch Otolaryngol Head Neck Surg.

[80] Labus J, Breil J, Stützer H, Michel O (2010). Meta-analysis for the effect of medical therapy vs. placebo on recovery of idiopathic sudden hearing loss. Laryngoscope.

[81] Wei BP, Stathopoulos D, O'Leary S (2013). Steroids for idiopathic sudden sensorineural hearing loss. Cochrane Database Syst Rev.

[82] Alexiou C, Arnold W, Fauser C, Schratzenstaller B, Gloddek B, Fuhrmann S, Lamm K (2001). Sudden sensorineural hearing loss: does application of glucocorticoids make sense?. Arch Otolaryngol Head Neck Surg.

[83] Egli Gallo D, Khojasteh E, Gloor M, Hegemann SC (2013). Effectiveness of systemic high-dose dexamethasone therapy for idiopathic sudden sensorineural hearing loss. Audiol Neurootol.

[84] Westerlaken BO, Stokroos RJ, Dhooge IJ, Wit HP, Albers FW (2003). Treatment of idiopathic sudden sensorineural hearing loss with antiviral therapy: a prospective, randomized, double-blind clinical trial. Ann Otol Rhinol Laryngol.

[85] Niedermeyer HP, Zahneisen G, Luppa P, Busch R, Arnold W (2003). Cortisol levels in the human perilymph after intravenous administration of prednisolone. Audiol Neurootol.

[86] Plontke SK, Girndt M, Meisner C, Probst R, Oerlecke I, Richter M, Steighardt J, Dreier G, Weber A, Baumann I, Plößl S, Löhler J, Laszig R, Werner JA, Rahne T (2016). Multizentrische Studie zur Hörsturztherapie – Planung und Konzeption. HNO.

[87] Bennett MH, Kertesz T, Perleth M, Yeung P, Lehm JP (2012). Hyperbaric oxygen for idiopathic sudden sensorineural hearing loss and tinnitus. Cochrane Database Syst Rev.

[88] Lawrence R, Thevasagayam R (2015). Controversies in the management of sudden sensorineural hearing loss: an evidence-based review. Clin Otolaryngol.

[89] Attanasio G, Covelli E, Cagnoni L, Masci E, Ferraro D, Mancini P, Alessandri E, Cartocci G, Filipo R, Rocco M (2015). Does the addition of a second daily session of hyperbaric oxygen therapy to intratympanic steroid influence the outcomes of sudden hearing loss?. Acta Otorhinolaryngol Ital.

[90] Cvorovic L, Jovanovic MB, Milutinovic Z, Arsovic N, Djeric D (2013). Randomized prospective trial of hyperbaric oxygen therapy and intratympanic steroid injection as salvage treatment of sudden sensorineural hearing loss. Otol Neurotol.

[91] Filipo R, Attanasio G, Viccaro M, Russo FY, Mancini P, Rocco M, Pietropaoli P, Covelli E (2012). Hyperbaric oxygen therapy with short duration intratympanic steroid therapy for sudden hearing loss. Acta Otolaryngol.

[92] Lamm H, Müller-Kortkamp C, Warnecke A, Pohl F, Paasche G, Lenarz T, Stolle SR (2016). Concurrent hyperbaric oxygen therapy and intratympanic steroid application as salvage therapy after severe sudden sensorineural hearing loss. Clin Case Rep.

[93] Parnes LS, Sun AH, Freeman DJ (1999). Corticosteroid pharmacokinetics in the inner ear fluids: an animal study followed by clinical application. Laryngoscope.

[94] Liebau A, Plontke SK (2015). Lokale Medikamententherapie bei Innenohrschwerhörigkeit. HNO.

[95] Salt AN, Plontke SK (2009). Principles of local drug delivery to the inner ear. Audiol Neurootol.

[96] Banerjee A, Parnes LS (2005). Intratympanic corticosteroids for sudden idiopathic sensorineural hearing loss. Otol Neurotol.

[97] Gianoli GJ, Li JC (2001). Transtympanic steroids for treatment of sudden hearing loss. Otolaryngol Head Neck Surg.

[98] Slattery WH, Fisher LM, Iqbal Z, Friedman RA, Liu N (2005). Intratympanic steroid injection for treatment of idiopathic sudden hearing loss. Otolaryngol Head Neck Surg.

[99] Burkart C, Linder T, Gärtner M (2013). Intratympanale Dexamethasongabe: Nutzen in der Behandlung des hochgradigen Hörsturzes. HNO.

[100] Mühlmeier G, Maier S, Maier M, Maier H (2015). Intratympanale Injektionstherapie bei therapierefraktärem Hörsturz: Eine sichere Therapieoption zur Sekundärtherapie. HNO.

[101] Crane RA, Camilon M, Nguyen S, Meyer TA (2015). Steroids for treatment of sudden sensorineural hearing loss: a meta-analysis of randomized controlled trials. Laryngoscope.

[102] Gao Y, Liu D (2016). Combined intratympanic and systemic use of steroids for idiopathic sudden sensorineural hearing loss: a meta-analysis. Eur Arch Otorhinolaryngol.

[103] Garavello W, Galluzzi F, Gaini RM, Zanetti D (2012). Intratympanic steroid treatment for sudden deafness: a meta-analysis of randomized controlled trials. Otol Neurotol.

[104] Lavigne P, Lavigne F, Saliba I (2016). Intratympanic corticosteroids injections: a systematic review of literature. Eur Arch Otorhinolaryngol.

[105] Li H, Feng G, Wang H, Feng Y (2015). Intratympanic steroid therapy as a salvage treatment for sudden sensorineural hearing loss after failure of conventional therapy: a meta-analysis of randomized, controlled trials. Clin Ther.

[106] Ng JH, Ho RC, Cheong CS, Ng A, Yuen HW, Ngo RY (2015). Intratympanic steroids as a salvage treatment for sudden sensorineural hearing loss? A meta-analysis. Eur Arch Otorhinolaryngol.

[107] Liebau A, Pogorzelski O, Salt AN, Plontke SK (2017). Hearing Changes After Intratympanically Applied Steroids for Primary Therapy of Sudden Hearing Loss: A Meta-analysis Using Mathematical Simulations of Drug Delivery Protocols. Otol Neurotol.

[108] Plontke SK, Meisner C, Caye-Thomasen P, Parnes LS, Agrawal S, Mikulec AA (2009). Intratympanic glucocorticoids for sudden sensorineural hearing loss. Cochrane Database Syst Rev.

[109] Battaglia A, Lualhati A, Lin H, Burchette R, Cueva R (2014). A prospective, multi-centered study of the treatment of idiopathic sudden sensorineural hearing loss with combination therapy versus high-dose prednisone alone: a 139 patient follow-up. Otol Neurotol.

[110] Günel C, Başal Y, Toka A, Eryılmaz A, Kurt Ömürlü I (2015). Efficacy of low-dose intratympanic dexamethasone for sudden hearing loss. Auris Nasus Larynx.

[111] Plontke SK, Liebau A, Pogorzelski O, Salt AN, Zenner HP Dependence of hearing improvement on the dose of intratympanically applied glucocorticoids: a meta-analysis using mathematical simulations of clinical drug delivery protocols.

[112] Plontke SK, Zimmermann R, Zenner HP, Löwenheim H (2006). Technical note on microcatheter implantation for local inner ear drug delivery: surgical technique and safety aspects. Otol Neurotol.

[113] Nguyen K, Kempfle JS, Jung DH, McKenna CE (2017). Recent advances in therapeutics and drug delivery for the treatment of inner ear diseases: a patent review (2011-2015). Expert Opin Ther Pat.

[114] Ayoob AM, Borenstein JT (2015). The role of intracochlear drug delivery devices in the management of inner ear disease. Expert Opin Drug Deliv.

[115] El Kechai N, Agnely F, Mamelle E, Nguyen Y, Ferrary E, Bochot A (2015). Recent advances in local drug delivery to the inner ear. Int J Pharm.

[116] Nakagawa T, Ito J (2011). Local drug delivery to the inner ear using biodegradable materials. Ther Deliv.

[117] Plontke SK, Glien A, Rahne T, Mäder K, Salt AN (2014). Controlled release dexamethasone implants in the round window niche for salvage treatment of idiopathic sudden sensorineural hearing loss. Otol Neurotol.

[118] Lambert PR, Carey J, Mikulec AA, LeBel C, Otonomy Ménièreʼs Study Group (2016). Intratympanic Sustained-Exposure Dexamethasone Thermosensitive Gel for Symptoms of Ménière's Disease: Randomized Phase 2b Safety and Efficacy Trial. Otol Neurotol.

[119] Suckfuell M, Canis M, Strieth S, Scherer H, Haisch A (2007). Intratympanic treatment of acute acoustic trauma with a cell-permeable JNK ligand: a prospective randomized phase I/II study. Acta Otolaryngol.

[120] Suckfuell M, Lisowska G, Domka W, Kabacinska A, Morawski K, Bodlaj R, Klimak P, Kostrica R, Meyer T (2014). Efficacy and safety of AM-111 in the treatment of acute sensorineural hearing loss: a double-blind, randomized, placebo-controlled phase II study. Otol Neurotol.

[121] Macleod MR, Michie S, Roberts I, Dirnagl U, Chalmers I, Ioannidis JP, Al-Shahi Salman R, Chan AW, Glasziou P (2014). Biomedical research: increasing value, reducing waste. Lancet.

[122] Chalmers I, Glasziou P (2009). Avoidable waste in the production and reporting of research evidence. Lancet.

[123] Plontke SK, Bauer M, Meisner C (2007). Comparison of pure-tone audiometry analysis in sudden hearing loss studies: lack of agreement for different outcome measures. Otol Neurotol.

[124] Burschka MA, Hassan HA, Reineke T, van Bebber L, Caird DM, Mösges R (2001). Effect of treatment with Ginkgo biloba extract EGb 761 (oral) on unilateral idiopathic sudden hearing loss in a prospective randomized double-blind study of 106 outpatients. Eur Arch Otorhinolaryngol.

[125] Rahne T, Buthut F, Plößl S, Plontke SK (2016). A software tool for pure-tone audiometry. Classification of audiograms for inclusion of patients in clinical trials. English version. HNO.

[126] Chen CY, Halpin C, Rauch SD (2003). Oral steroid treatment of sudden sensorineural hearing loss: a ten year retrospective analysis. Otol Neurotol.

[127] Rauch SD, Halpin CF, Antonelli PJ, Babu S, Carey JP, Gantz BJ, Goebel JA, Hammerschlag PE, Harris JP, Isaacson B, Lee D, Linstrom CJ, Parnes LS, Shi H, Slattery WH, Telian SA, Vrabec JT, Reda DJ (2011). Oral vs intratympanic corticosteroid therapy for idiopathic sudden sensorineural hearing loss: a randomized trial. JAMA.

[128] Wu W, Thuomas KA (1995). MR imaging of 495 consecutive cases with sensorineural hearing loss. Acta Radiol.

[129] Daniels RL, Swallow C, Shelton C, Davidson HC, Krejci CS, Harnsberger HR (2000). Causes of unilateral sensorineural hearing loss screened by high-resolution fast spin echo magnetic resonance imaging: review of 1,070 consecutive cases. Am J Otol.

[130] Schick B, Brors D, Koch O, Schäfers M, Kahle G (2001). Magnetic resonance imaging in patients with sudden hearing loss, tinnitus and vertigo. Otol Neurotol.

[131] Carrier DA, Arriaga MA (1997). Cost-effective evaluation of asymmetric sensorineural hearing loss with focused magnetic resonance imaging. Otolaryngol Head Neck Surg.

[132] Aarnisalo AA, Suoranta H, Ylikoski J (2004). Magnetic resonance imaging findings in the auditory pathway of patients with sudden deafness. Otol Neurotol.

[133] Newton JR, Shakeel M, Flatman S, Beattie C, Ram B (2010). Magnetic resonance imaging screening in acoustic neuroma. Am J Otolaryngol.

[134] Sheppard IJ, Milford CA, Anslow P (1996). MRI in the detection of acoustic neuromas--a suggested protocol for screening. Clin Otolaryngol Allied Sci.

[135] Dawes PJ, Jeannon JP (1998). Audit of regional screening guidelines for vestibular schwannoma. J Laryngol Otol.

[136] Ramos HV, Barros FA, Yamashita H, Penido Nde O, Souza AC, Yamaoka WY (2005). Magnetic resonance imaging in sudden deafness. Braz J Otorhinolaryngol.

[137] Fitzgerald DC, Mark AS (1998). Sudden hearing loss: frequency of abnormal findings on contrast-enhanced MR studies. AJNR Am J Neuroradiol.

[138] Stokroos RJ, Albers FW, Krikke AP, Casselman JW (1998). Magnetic resonance imaging of the inner ear in patients with idiopathic sudden sensorineural hearing loss. Eur Arch Otorhinolaryngol.

[139] Staecker H, Rodgers B (2013). Developments in delivery of medications for inner ear disease. Expert Opin Drug Deliv.

